# Extensive sequence and structural evolution of Arginase 2 inhibitory antibodies enabled by an unbiased approach to affinity maturation

**DOI:** 10.1073/pnas.1919565117

**Published:** 2020-07-02

**Authors:** Denice T. Y. Chan, Lesley Jenkinson, Stuart W. Haynes, Mark Austin, Agata Diamandakis, Daniel Burschowsky, Chitra Seewooruthun, Alexandra Addyman, Sebastian Fiedler, Stephanie Ryman, Jessica Whitehouse, Louise H. Slater, Ellen Gowans, Yoko Shibata, Michelle Barnard, Robert W. Wilkinson, Tristan J. Vaughan, Sarah V. Holt, Vincenzo Cerundolo, Mark D. Carr, Maria A. T. Groves

**Affiliations:** ^a^Cancer Research UK–AstraZeneca Antibody Alliance Laboratory, CB21 6GP Cambridge, United Kingdom;; ^b^Antibody Discovery & Protein Engineering, BioPharmaceuticals Research & Development, AstraZeneca, CB21 6GH Cambridge, United Kingdom;; ^c^Leicester Institute of Structural and Chemical Biology, University of Leicester, LE1 7HB Leicester, United Kingdom;; ^d^Department of Molecular and Cell Biology, University of Leicester, LE1 7HB Leicester, United Kingdom;; ^e^Early Oncology Discovery, Oncology Research & Development, AstraZeneca, CB21 6GH Cambridge, United Kingdom;; ^f^Medical Research Council Human Immunology Unit, Weatherall Institute of Molecular Medicine, University of Oxford, OX3 9DS Oxford, United Kingdom

**Keywords:** affinity maturation, Arginase 2, inhibitory antibodies, antibody engineering, ribosome display

## Abstract

Affinity maturation is a powerful technique in antibody engineering for the in vitro evolution of antigen binding interactions. Key to the success of this process is the expansion of sequence and combinatorial diversity to increase the structural repertoire from which superior binding variants may be selected. However, conventional strategies are often restrictive and only focus on small regions of the antibody at a time. In this study, we used a method that combined antibody chain shuffling and a staggered-extension process to produce unbiased libraries, which recombined beneficial mutations from all six complementarity-determining regions (CDRs) in the affinity maturation of an inhibitory antibody to Arginase 2 (ARG2). We made use of the vast display capacity of ribosome display to accommodate the sequence space required for the diverse library builds. Further diversity was introduced through pool maturation to optimize seven leads of interest simultaneously. This resulted in antibodies with substantial improvements in binding properties and inhibition potency. The extensive sequence changes resulting from this approach were translated into striking structural changes for parent and affinity-matured antibodies bound to ARG2, with a large reorientation of the binding paratope facilitating increases in contact surface and shape complementarity to the antigen. The considerable gains in therapeutic properties seen from extensive sequence and structural evolution of the parent ARG2 inhibitory antibody clearly illustrate the advantages of the unbiased approach developed, which was key to the identification of high-affinity antibodies with the desired inhibitory potency and specificity.

## Significance

We describe an antibody optimization strategy that seeks to overcome the restrictive nature of existing affinity-maturation methods, by rapidly exploring a vast sequence space in an unbiased manner through application of PCR techniques and ribosome display. We exemplified the significance of this method by contrasting the crystal structure of the parent and optimized antibodies bound to Arginase 2, which revealed a striking reorientation of the binding paratope, concurrent with distinct improvements in inhibitory potency and binding properties. The nature and magnitude of the epitope expansion was extraordinary and unlikely to have been produced through conventional affinity-maturation methods. This innovative approach demonstrates broad applicability to the optimization of candidate therapeutic antibodies, even those less amenable to CDRH3 targeting.

In antibody engineering, affinity maturation is a method of directed molecular evolution used to improve the affinity and binding interactions of an antibody to its antigen. This is often done to fulfill the required potency of biotherapeutics in vivo. In the natural antibody maturation process in B cells, Ig genes undergo a diversification of sequences in the variable segments via somatic hypermutation, followed by a selection of high-affinity binders by clonal selection (1). In vitro affinity maturation mimics this process through the introduction of sequence diversity into a candidate antibody to produce libraries of mutational variants, and subsequent selections using display methods, such as phage or ribosome display, to find higher-affinity binders. Key to the success of these processes is the initial expansion of sequence and consequently structural diversity, to produce a library from which superior binders can be found. Studies of affinity maturation have shown that apart from mutations that allow for formation of favorable hydrogen bonds, electrostatic interactions, and van der Waals contacts, large conformational changes are often required as a mechanism for preorganizing or reorientating the antibody paratope to improve shape complementarity to the antigen (2–4). Hence, a fundamental objective of in vitro affinity maturation is to design strategies that could maximize the mutational and combinatorial diversity in a given library, using a variety of mutagenesis and recombination techniques.

Phage display is commonly used to optimize sequences in the complementarity-determining regions (CDRs) of an antibody. Only small numbers of residues are normally targeted for mutagenesis at a time, due to limitations in transformation efficiency (5). However, mutations in single CDRs are often insufficient, and synergistic mutations from different CDRs may be required to bring about substantial affinity gains. One way to connect such mutations is via recombination of selection outputs, which has been shown as a successful method in extending the affinity and potency gains achievable from the optimization of single CDRs (6–9). Typically, recombination of only two CDRs, usually one from the variable heavy (VH) and one from the variable light (VL) region, is considered at a time for sufficient coverage within the library size limitations of phage display. Ribosome display does not require a bacterial transformation step and can theoretically cover populations of over 10^12^ in size (9, 10). It is therefore feasible to use ribosome display to select populations of larger sizes to cover libraries of greater diversity. Indeed, it has been shown that recombination libraries selected using ribosome display have the advantage of greater sequence and structural diversity compared to phage display (11), which affords a greater chance of finding improved binders.

With the greater capacity of ribosome display, it is possible to consider more ambitious library builds. We envisaged an approach in which advantageous mutations from all six CDRs could be allowed to recombine freely in an unbiased manner. Here we describe a strategy which utilizes antibody chain shuffling and the staggered-extension process (StEP) to create such libraries.

Antibody chain shuffling involves a repairing of heavy- and light-chain repertoires in a population of antibody variants. Usually this involves the recombination of one or more heavy or light chains of a particular antibody, with a library of heavy or light chains, using standard molecular biology techniques. It is a method that exploits chain promiscuity and is useful in increasing the combinatorial diversity of antibody libraries (12–14). In this study, we employed this method to shuffle large populations of optimized VH and VL sequences simultaneously. StEP recombination is a method similar to DNA shuffling, based on template switching during polymerase-catalyzed primer extension. It was first demonstrated as an in vitro recombination technique for enzyme evolution, in which mutational variants of subtilisin E were recombined to improve thermostability (15). It has since been used to engineer other enzymes and proteins with improved or novel functions (16) and has also been used to produce chimeric variants of five single-domain antibody (sdAb) templates to study the regions responsible for its binding properties (17). The StEP recombination technique utilizes a modified PCR protocol with very short annealing and extension steps to generate partial, “staggered” DNA fragments, which are then able to prime to and extend on a different template during subsequent annealing cycles (16, 18). This promotes cross-over events along the full length of the templates to produce a library of chimeric constructs of the parental population. For effective priming and cross-over to occur, a degree of sequence homology is required between the starting templates, so mutational libraries of single-chain variable fragments (scFv) with a similar framework and diverse CDR sequences are an ideal template for this method. An added advantage of StEP recombination is that the point of cross-over is random and can occur at any point of homology along the scFv, hence it would be possible to have recombination between the CDRs of the same chain, so called “intrachain” recombination.

Pool maturation is another similar strategy that seeks to lift some of the restrictions of conventional antibody optimization processes (19). While chain-shuffling and StEP recombination are used for retaining and recombining all of the potentially synergistic mutations in all of the CDRs, pool maturation is the simultaneous optimization of multiple leads of interest, rather than just one. In this study, we pooled seven leads identified during screening of the recombined outputs and introduced further diversity to the pool through error-prone (EP) mutagenesis in a single library, from which higher-affinity leads were selected using ribosome display. This granted a fresh chance to reexplore and improve on the potential of interesting leads.

**Fig. 1.**
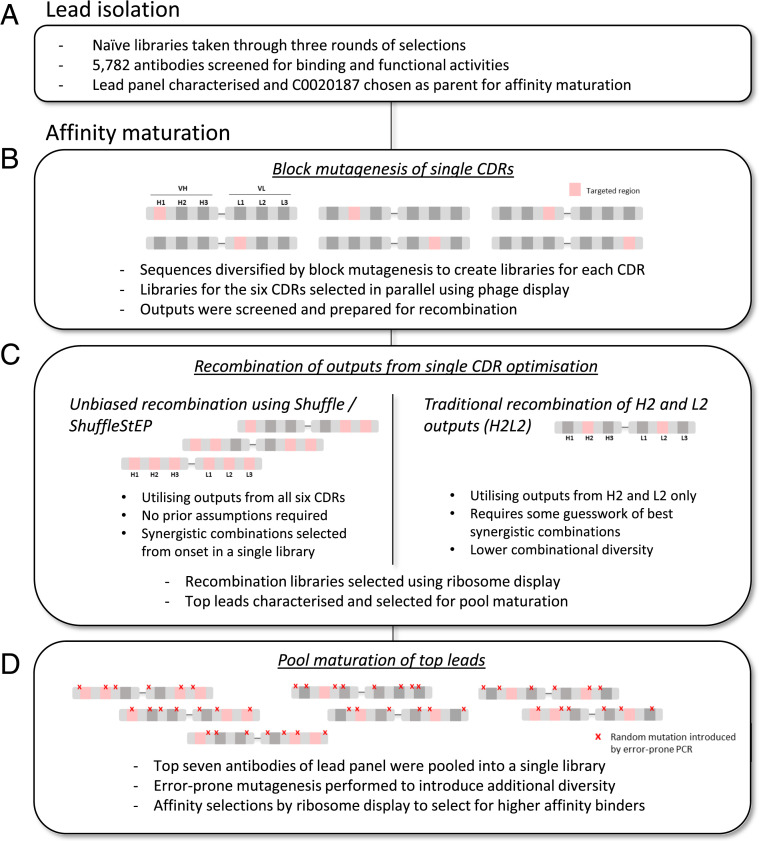
Overview of the antibody discovery cascade. (*A*) Parental lead C0020187 was isolated through phage-display selections of naïve libraries and subsequent binding and functional screens. It was then taken through an extensive affinity-maturation campaign, which involves (*B*) the targeted mutagenesis of single CDRs, (*C*) two parallel strategies for recombination, and (*D*) a final pool maturation of the top leads.

In this study, we utilized these methods to generate a panel of high-affinity antibodies to human Arginase 2 (ARG2). C0020187 is a human monoclonal antibody specific for ARG2 that was derived from phage-display selections on naïve libraries of scFvs based on human Ig variable regions (20). We wanted to improve the affinity and potency of C0020187 to enhance its efficacy in vivo. To achieve this, we sought to affinity-mature C0020187 in an unbiased optimization campaign. This included a Shuffle/ShuffleStEP method, which optimized and recombined mutations accumulated in all six CDRs, followed by a pool-maturation method that simultaneously affinity-matured a panel of seven antibodies (Fig. 1). This resulted in final therapeutic candidates that showed considerably improved binding affinity and increased enzyme inhibition potency, as well as more idealized binding properties resulting from an apparent relief from negative cooperativity of binding. Extensive sequence and structural changes were observed in the lead antibodies as they evolved through the affinity-maturation process, which resulted in a large epitope shift enabling increases in contact surface and shape complementarity. The dramatic changes and improvements observed were clearly greatly facilitated by the unbiased and inclusive approach to affinity maturation developed and would almost certainly not have been achieved by conservative traditional methods. The innovative approach reported here promises a widely applicable step change in our ability to optimize the affinity and potency of potential therapeutic antibodies.

## Results

### Optimization of Sequences in the Six CDR Regions.

As the initial step of affinity maturation for antibody C0020187, block mutagenesis was used to diversify sequences in the CDR regions. Multiple libraries were designed for optimal coverage of sequences in each CDR (Fig. 1*B* and *SI Appendix*, Fig. S1). All six CDRs were included in the mutagenesis scheme and optimized in parallel to maximize the sequence space from which variants with improved affinities may emerge, and later recombined.

**Fig. 2.**
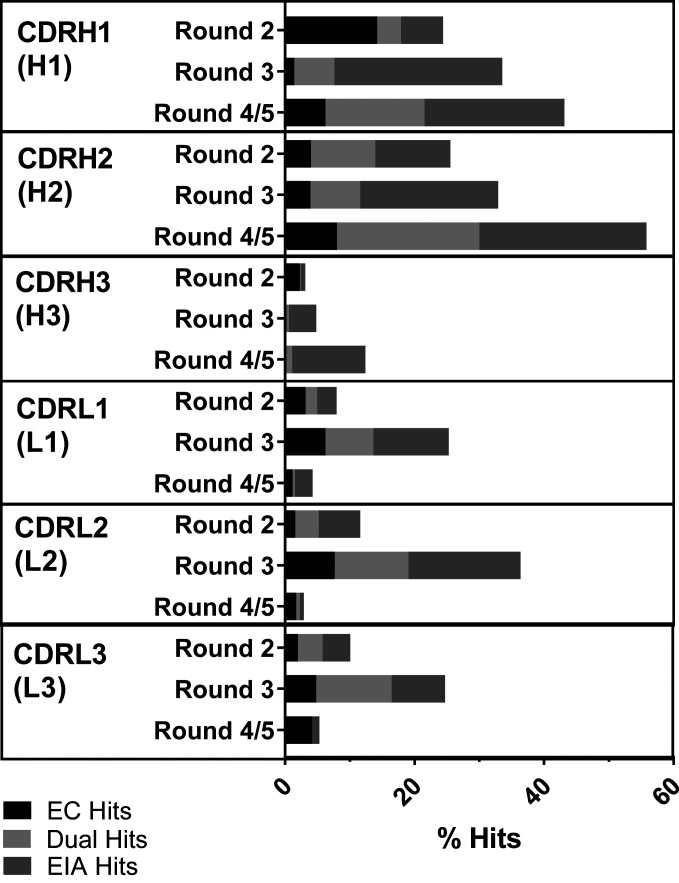
Hit rates from the outputs of CDR optimization. The outputs from each selection round for each CDR were prepared as crude scFvs in periplasmic extracts in a 96-well format and screened using the EC and EIA assays. The parental antibody C0020187 was used as a benchmark in both assays, against which significantly improved clones were identified as hits. The percentage hit rates (number of hits/number screened × 100) for the outputs from each CDR are represented in a bar graph for each round. Each bar is further broken down to indicate whether they were EC hits, EIA hits, or hits in both assays (dual hits).

These libraries were selected in soluble phage-display selections. Individual clones were sampled from the outputs of each CDR group, and tested as scFvs in periplasmic extracts (21) using a homogeneous time-resolved fluorescence-based epitope competition (EC) assay (22) in parallel with a functional screen, the enzyme inhibition assay (EIA). The EC assay is a measure of how well an incoming scFv is able to compete with the parental antibody for the epitope and is thus a surrogate measure for binding affinity. The EIA assay is a read-out of functional activity, benchmarked against the parental antibody’s ability to inhibit the enzymatic activity of ARG2. Together, these assays inform which optimization groups were likely to generate clones with improved affinity and potency over the parent. Optimization in the CDRH1 (H1) and CDRH2 (H2) groups presented with the highest total hit rates, at 43% and 54%, respectively, in the final rounds, whereas CDRH3 (H3) optimization resulted in the lowest hit rates at 12% (Fig. 2). The total hit rate for CDRL2 (L2) at round 3 is 36%, compared to 25% for CDRL1 (L1) and CDRL3 (L3). Interestingly, the hit rates for the VL outputs decreased after round 3, suggesting that there was no further enrichment of higher-affinity clones in these selection groups at the lowest antigen concentration bracket.

### Unbiased Recombination of Optimized CDR Regions Using the Shuffle and ShuffleStEP Methods.

The unbiased recombination libraries were built in a process of VH/VL chain-shuffling with or without the enhancement of StEP recombination (Fig. 1*C* and *SI Appendix*, Fig. S2). Chain-shuffling was carried out through the amplification of the VH-optimized phage-display selection outputs (which targeted H1, H2, and H3) and VL-optimized selection outputs (which targeted L1, L2, and L3), followed by a recombination PCR, which assembled them into full scFv constructs via an overlapping linker. The result was a recombined Shuffle library that paired optimized sequences from one of the three VH CDRs and one of the three VL CDRs at random. Sequencing of the Shuffle library variants showed that mutations were incorporated fairly evenly from each CDR, with a high recombination frequency of 90% (Table 1). The remaining 10% carried mutations in one CDR. To promote additional recombination events, the Shuffle library was used as a base template for StEP recombination to produce the ShuffleStEP library. Template-swapping occurs at random points along the length of the scFv construct during the very short annealing and extension StEP amplification cycles, producing chimeric constructs of the template population. This also resulted in a library with broadly similar mutational frequency across the CDRs, with a recombination rate of 81%. Of the remaining, 16% carried mutations in one CDR, and a small percentage of parental sequence (3%) was observed. This suggested that there was likely some back-crossing of the nonmutated regions of the base templates, which is intriguing as such clones can only arise through a physical recombination event.

**Table 1.** Sequence summary of the Shuffle and ShuffleStEP libraries

			Mutational frequency (%)							
Library	Round	[ARG2] (nM)	H1	H2	H3	L1	L2	L3	Recombination frequency (%)	CDR diversity (%)
Shuffle	0	N/A	32	43	32	30	44	33	90	100
ShuffleStEP		N/A	34	39	30	28	38	33	81	100*
										
Shuffle	2	5.0	63	43	3	13	43	35	70	100
ShuffleStEP		5.0	63	38	0	13	43	35	70	100
										
Shuffle	3	1.0	65	43	13	28	23	40	85	93
ShuffleStEP		1.0	64	51	8	23	33	28	74	100
										
Shuffle	3	0.5	50	65	5	13	33	63	85	90
ShuffleStEP		0.5	60	45	7	21	29	43	69	93

The table shows the abundance of clones with mutations in each CDR, as well as the recombination frequency and CDR amino acid diversity for each round. Mutational frequency is calculated by dividing the number of clones with amino acid mutations in the designated CDR by the number of clones sampled × 100. Recombination frequency was defined as the percentage of clones in the population with recombined mutations in different CDRs (number of clones with amino acid mutations in more than one CDR/number of clones sampled × 100). CDR diversity describes the percentage of clones in the population with a unique CDR sequence combination (number of clones with a unique CDR amino acid sequence combination/number of clones sampled × 100). The concentration of ARG2 used in each selection round is also indicated.

* Excluding 2.5% parent.

Since the point of cross-over is random and not restricted at the linker region during the StEP recombination reaction, it is thus possible for intrachain recombination (i.e., recombination of mutations within the same VH or VL chain) to occur. Based on sequencing data, 23% of sequences showed intrachain recombination in the ShuffleStEP library, compared to 12% of sequences in the Shuffle library. It was somewhat unexpected that intrachain recombination should occur in the Shuffle library, and this result led us to consider that a level of swapping may take place between sequence-similar templates during the PCR-based reaction used in chain-shuffling. Such template-swapping activity also gave rise to library variants with recombination between three or more CDRs, further increasing combinatorial diversity (*SI Appendix*, Table S1).

### Selection of Improved Variants from the Shuffle and ShuffleStEP Libraries Using Ribosome Display.

The Shuffle and ShuffleStEP libraries were taken through three rounds of ribosome display selections to isolate binders of higher affinity. Antigen concentration was lowered at each subsequent round to increase stringency so that higher-affinity binders are preferentially selected. The selection outputs were monitored by sequencing of round 2 and 3 outputs, with a sequence summary shown in Table 1. Mutational frequency was broadly similar among the six CDRs in the starting libraries (round 0), as expected since the same amount of template from each single-CDR optimized output was used in library building. As the selections proceeded, there was a large decrease in the number of clones with mutations in H3, reducing from 32% and 30% to 3% and 0% for the Shuffle and ShuffleStEP libraries, respectively, at round 2. This confirms that clones carrying mutations in H3 are not well-tolerated and rapidly outselected, which mirrors the universally poor hit rates for H3-mutated outputs during the single-CDR-targeted phage-display selections shown previously. Mutation rates in H3 rose slightly again to 5 to 7% in round 3 (0.5 nM), but these were mostly single amino acid mutations that were randomly incorporated during the numerous PCR amplification rounds of ribosome display. There was a general increase in the percentages of clones carrying mutations in H1 and H2. Of the VL CDRs, L3 and L1 present with the highest and lowest number of clones carrying mutations after three rounds of selection, respectively. The overall CDR amino acid sequence diversity remains high, not dropping below 90% throughout the selection rounds.

**Fig. 3.**
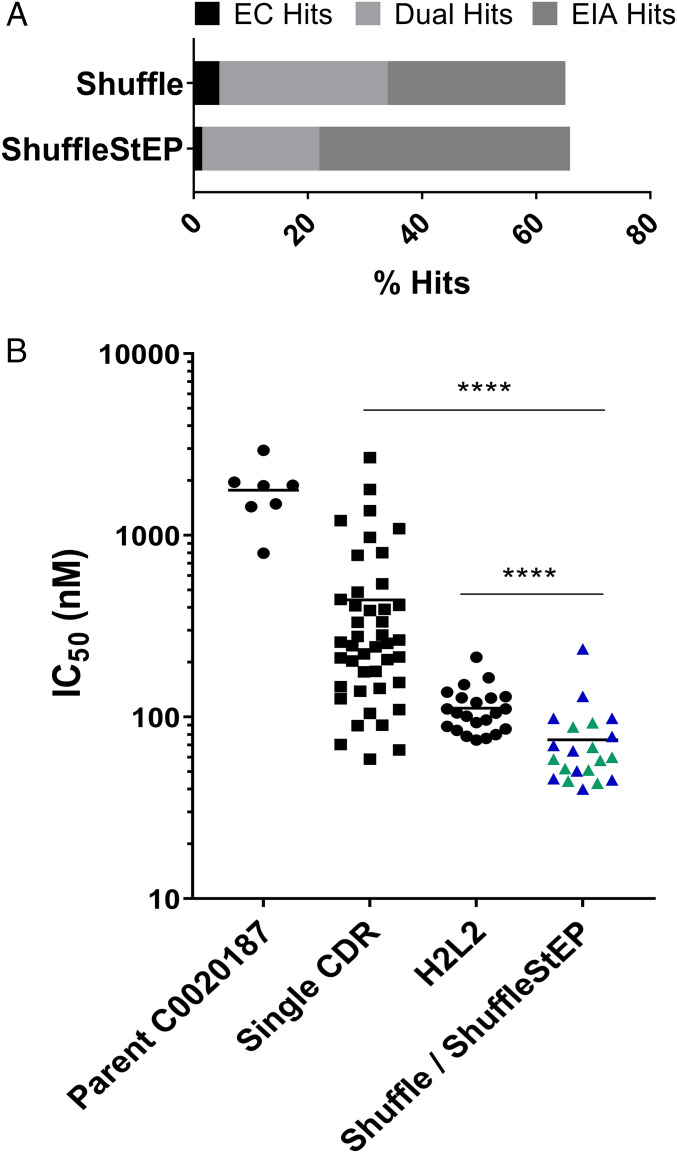
Screening of the Shuffle and ShuffleStEP libraries. (*A*) Percentage hit rates (number of hits/number screened × 100) for the Shuffle and ShuffleStEP libraries in the crude scFv screen. (*B*) A comparison of IC_50_ values for the purified scFvs originating from the Shuffle (blue), ShuffleStEP (green), H2L2, and the single-CDR mutated libraries in the EC screen compared to parent C0020187. Each data point represents the average IC_50_ value of an antibody candidate from at least two independent tests, and the data points for parental C0020187 shows the average of measurements of different batches across seven independent experiments. A two-tailed Mann–Whitney *U* test was used to calculate statistical significance: ****P < 0.0001.

The Shuffle and ShuffleStEP round 2 and round 3 outputs were screened as crude scFvs in periplasmic preparations and tested for their ability to bind and inhibit ARG2 in the EC and EIA assays, respectively. A total of 660 clones were screened for each library, resulting in a similar total hit rate of 65% for both libraries (Fig. 3*A*). Sequence-diverse hits from the crude single-chain screen (21 candidates) were purified and titrated to determine IC_50_ values in the EC assay (Fig. 3*B*). Leads from the Shuffle/ShuffleStEP optimization performed significantly better than the parental antibody. Compared to hits from the single-CDR optimisations, which had a wide spread of IC_50_ values, the leads from the Shuffle/ShuffleStEP library showed a general trend of lower IC_50_ values, suggesting that the process may have enriched for clones with higher-affinity binding. As a benchmark to the Shuffle/ShuffleStEP strategy, a more traditional method involving the recombination of two CDRs, in this case H2 and L2, was also carried out (described in Fig. 1*C*). The library was built using recombination PCR and selected similarly to the Shuffle/ShuffleStEP library using ribosome display. Screening in the EC assay suggested that the Shuffle/ShuffleStEP leads also showed a lower trend in IC_50_ values than the leads from H2L2 (Fig. 3*B*), suggesting there may be an enrichment of better binders using the Shuffle/ShuffleStEP strategy.

### Pool Maturation of Top Antibody Candidates.

In a final effort to improve binding affinities, the top seven leads that performed best in the screening cascade were chosen for further optimization using a pool-maturation approach (Fig. 1*D*). The scFv constructs of these leads were combined in equal amounts and EP mutagenesis was applied to diversify their sequences as a pool. Two consecutive rounds of EP mutagenesis were used to introduce mutations at random throughout the scFv regions, under conditions that typically result in an average of 8 amino acid changes per scFv construct. The EP library was then taken through five rounds of soluble affinity selection, with decreasing concentration of ARG2 from 6 nM to 20 pM. The outputs were subcloned, and 1,736 clones were screened as periplasmic preparations in the EC and EIA assays. In this screen, 234 hits were identified and sequenced. Based on sequence diversity and hit values, 17 clones were converted to Fab by subcloning of VH and VL domains into vectors expressing human Fab constant regions (23) and ranked by affinity to human ARG2.

### Optimized Antibodies Show Substantial Improvements in Binding Properties and Inhibition Potency.

**Fig. 4.**
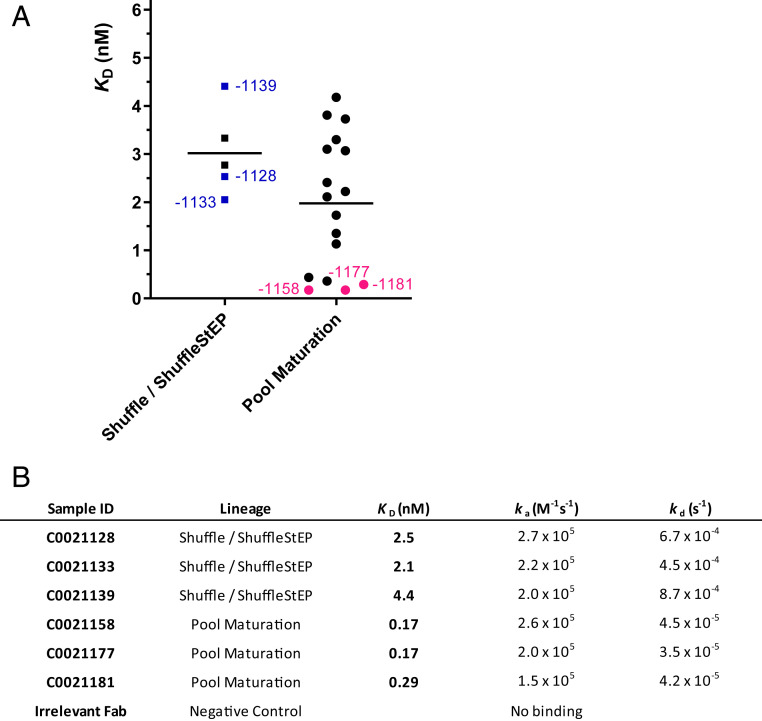
Binding affinities of affinity-matured antibodies to human ARG2. (*A*) A graphic comparison of estimated *K*_D_ values of antibodies derived from the Shuffle/ShuffleStEP and pool-maturation strategies. Representative leads from the panel, which are further discussed here, are annotated in blue and pink for the Shuffle/ShuffleStEP stream and pool-maturation stream respectively. (*B*) Kinetic parameters of the representative leads derived on the Octet software. *k*_a_: association rate constant; *k*_d_: dissociation rate constant; *K*_D_: equilibrium dissociation constant.

Binding affinities of the affinity-matured antibodies to human ARG2 were measured by biolayer interferometry (BLI). ARG2 was used as the ligand and Fabs were titrated as the analyte. Fabs from the Shuffle/ShuffleStEP recombination C0021128, C0021133, and C0021139 bound to human ARG2 with a *K*_D_ range of 2 to 4 nM (Fig. 4). Further improvements were observed after pool maturation, and the top three antibodies, C0021158, C0021177, and C0021181 bound to human ARG2 with *K*_D_ values of 0.1 to 0.3 nM.

While attempting to measure the affinity of the parental antibody, it was observed that the sensorgrams of C0020187 did not fit well to the classic 1:1 binding model, exhibiting multiphasic binding interactions and less satisfactory fits than its affinity-matured progeny (*SI Appendix*, Fig. S3). Moreover, the binding signals of the parental antibody were approximately two- to threefold lower than the affinity-matured antibodies. These observations coincided with several notable characteristics of the behavior of the parental antibody that are suggestive of negativity cooperativity on binding to trimeric ARG2. Rationalizing that trimeric ARG2 has potentially three distinct antibody binding sites, we performed size-exclusion chromatography (SEC) analysis to establish the ratio of Fab to ARG2 upon complex formation. Interestingly, Fab C0020187 appeared to bind ARG2 as a tight 1:3 complex in solution, equivalent to one Fab molecule per trimer, whereas the optimized Fabs C0021158, C0021177, and C0021181 formed tight 3:3 complexes with ARG2 as would be expected if all three sites were equivalent (*SI Appendix*, Fig. S4). Taken together, these observations suggest significant negative cooperativity for the parental antibody’s interaction with trimeric ARG2, an issue that appears to have been resolved through the antibody optimization process.

To enable assessment of affinity improvements gained during antibody optimization, we fitted C0020187 binding data to a model that allows the resolution of two *K*_D_ values, such that the data be reflective of a high-affinity binding site for the association of the one Fab per trimer of ARG2, and a low-affinity binding site for binding of additional Fabs. While this model is not able to accurately model the nonidealized three-site binding of Fab to ARG2, it should give us a reasonable measure of the initial high-affinity binding site, which would be the dominant interaction captured in the concentration range used in the BLI experiments. This analysis produced a much better fit than the 1:1 binding model (*SI Appendix*, Fig. S3), suggesting a *K*_D_ value of ∼10 nM for the initial high-affinity binding site, and a substantially weaker binding interaction, which is tentatively estimated to be in the low micromolar range for the low-affinity binding sites. These affinity values would be consistent with the differences in the observed stoichiometry of the Fab:ARG2 complex between SEC and the crystal structure, because Fabs binding to sites with low micromolar affinity would not be expected to remain bound during SEC analysis, but would be present at the concentrations used to obtain crystals of the complex.

**Fig. 5.**
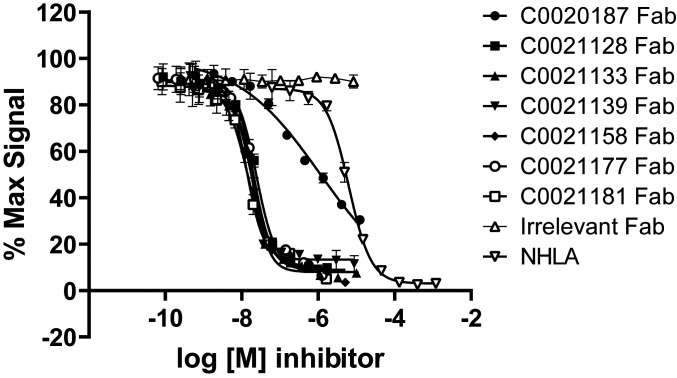
Affinity-matured antibodies show enhanced enzymatic inhibition of recombinant human ARG2. Recombinant human ARG2 (1 µg/mL, ∼24 nM) was preincubated for 2 h with titrations of the lead antibodies expressed as Fab fragments alongside an irrelevant Fab fragment and the small-molecule ARG1/ARG2 inhibitor NHLA (24). The enzymatic reaction was initiated by the addition of arginine (25 mM) and allowed to proceed for 1 h at room temperature, after which the reaction was stopped and the concentration of urea was measured colorimetrically. The data were fitted using a nonlinear regression curve fit. The graph depicts a representative dataset. Two independent experiments resulted in mean IC_50_ values for NHLA of 5.6 µM ± 0.7 µM and for C0020187 of 0.9 µM ± 0.2 µM. The remaining affinity-matured Fabs all reached the detection limit of the assay, which puts their IC_50_ values at less than or equal to ∼15 nM.

Potent inhibition of the enzymatic activity of ARG2 is a crucial property of candidate therapeutic antibodies. Our antibodies showed inhibition to recombinant human ARG2 as scFvs in the primary screens. We hereby demonstrate that our antibodies also show potent inhibition of human ARG2 in Fab format (Fig. 5). The parental antibody, C0020187, showed inhibition to human ARG2 with an IC_50_ of ∼890 nM, with an inhibition curve that lies to the left of the small-molecule inhibitor NHLA (NG-hydroxy-ʟ-arginine [Millipore]) (24). The affinity-matured antibodies all showed substantially more potent inhibition than either the parental antibody or NHLA, with inhibition curves that are further left-shifted compared to the parent. However, it was not possible to obtain accurate IC_50_ values for the affinity-matured antibodies, as the assay has reached its limit of sensitivity, with apparent IC_50_ values equivalent to or below half the enzyme concentration required in the assay (∼12 nM). Attempts to reduce the enzyme concentration used in the assay resulted in an unacceptable loss in signal window.

Due to the apparent negative cooperativity in binding of the parental antibody, as described above for the BLI and SEC analyses, and the limited sensitivity of the enzyme inhibition assay, it is difficult to determine a definitive measure of the improvement that resulted from the affinity-maturation process. However, the assay results suggest that the final lead antibody is at least 50-fold more potent than the parent, with an IC_50_ of below 15 nM. This improvement is in line with the estimated affinity improvements from parent to lead antibody of ∼50-fold in going from *K*_D_ ∼10 nM (high-affinity interaction) of the parent to 0.17 nM for the optimized antibodies.

### Evolution of Extensive Sequence and Structural Changes during Antibody Optimization.

**Fig. 6.**
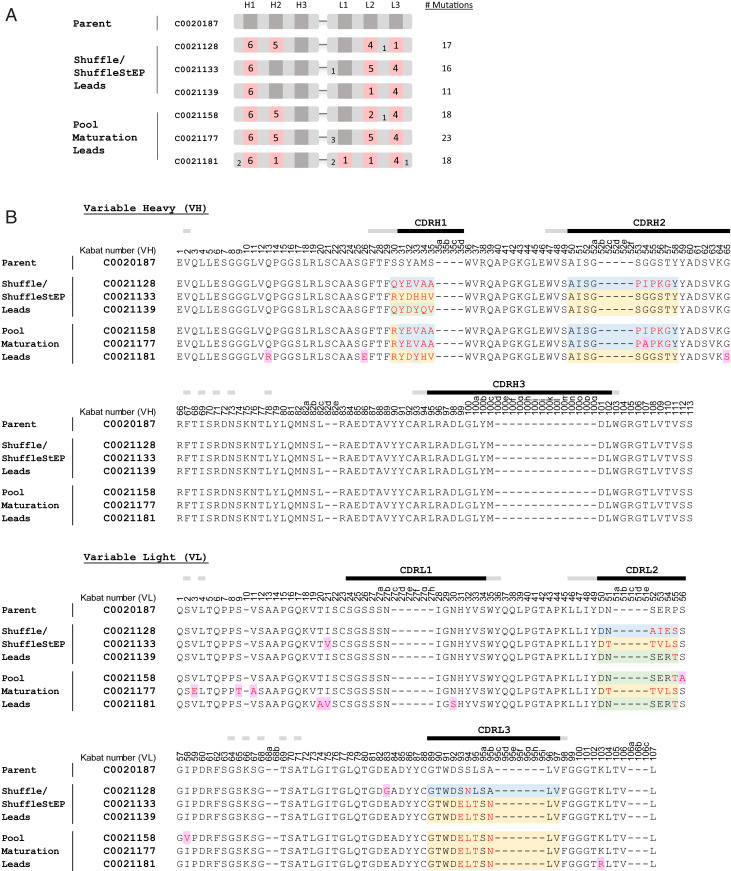
Sequence comparison of optimized antibodies with the parent. (*A*) A schematic showing the position and number of residues which have been mutated in each affinity-matured lead. Mutated CDRs are shown in pink. The number of mutations in each region is annotated on the blocks. (*B*) The amino acid sequence alignment of the affinity-matured leads to the parent C0020187. The residues are annotated according to Kabat numbering (25). CDR regions are marked with black bars, with flanking Vernier residues marked with gray. The residues that differ from the parent in the affinity-matured antibodies are in red type. Sequences in selected targeted regions that differ between the Shuffle, ShuffleStEP, and pool-maturation leads are highlighted in blue, yellow, and green based on sequence similarity to each other. Amino acids that were likely to have arisen through EP mutagenesis or spontaneous mutation (as they were not targeted in the mutagenesis scheme or descended from a parental construct) are highlighted in purple.

Comparison of the amino acid sequences of the optimized antibodies to the parent revealed extensive sequence evolution during the affinity-maturation process. This is illustrated by the sequence alignment and schematic highlighting the mutational changes which occurred in the top leads from the Shuffle/ShuffleStEP and pool-maturation streams in Fig. 6*A*. The leads produced from the Shuffle/ShuffleStEP recombination carry a high number of mutations, ranging from 11 to 17 amino acid changes per scFv. The mutations were present across three to four CDRs, which exemplifies the products of intrachain recombination, produced through template swapping to create recombination within the same chain. Further sequence diversity is seen in the pool maturation leads, which have a range of 18 to 23 mutations per scFv, across four to five CDRs. The scattering of single-amino acid mutations throughout the constructs also adds to the diversity (Fig. 6*B*), likely introduced by EP mutagenesis, or spontaneous mutations, which arise from in vitro amplification, during the ribosome display selection and recovery process.

To understand the lineage of the pool-maturation leads, we examined the sequences to determine from which parental construct each lead has evolved. Surprisingly, the pool-maturation leads did not appear to have evolved from a single parent but contained hybrid elements of various templates in the starting library (Fig. 6*B*). For example, C0021158 contained H1 and H2 sequences most closely related to C0021128, L2 sequences derived from C0021139, and L3 sequences that were similar to C0021133 or C0021139. Antibody C0021177 also contained VH sequences that were most closely related to C0021128, but its VL sequences most closely resembled C0021133. The VH of C0021181 mostly resembled C0021133, but its L2 sequences were most likely derived from C0021139. The extent of such sequence shuffling is very intriguing. It is possible that some of these mutations may have arisen through the EP process and were then serendipitously selected for, but such hybrid sequences were also observed in the starting library. This supports the earlier observation that template swapping may occur spontaneously and be propagated during ribosome display selections, creating unexpected combinatorial diversity.

**Fig. 7.**
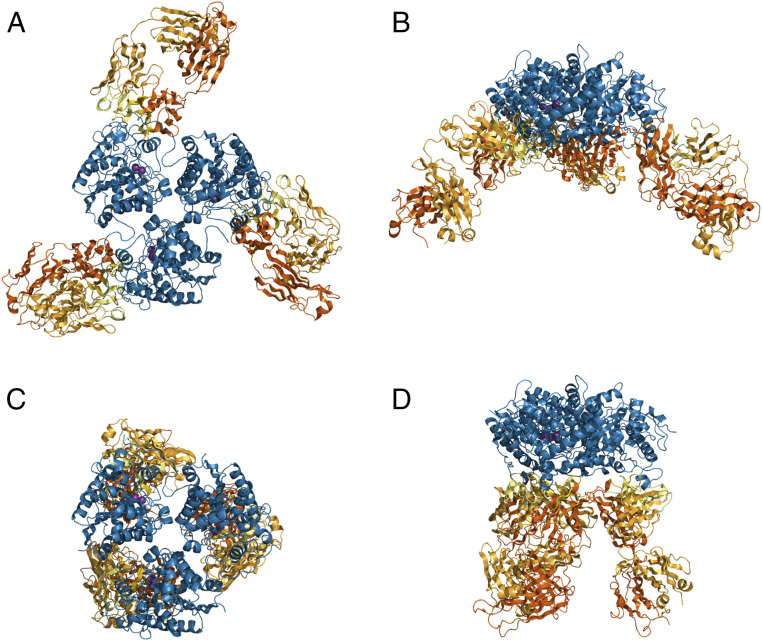
Structural overviews of ARG2 bound to two inhibitory antibodies (Fabs), comparing the parent inhibitory antibody (C0020187) to an affinity-matured therapeutic lead (C0021158). (*A*) ARG2 trimer (blue cartoon) bound to Fab C0020187 and (*C*) to Fab C0021158 (PDB ID code 6SS2) (VH/CH in orange and VL/CL in light orange, CDRs in yellow and light yellow for VH and VL, respectively) shown in “top” view. In the active sites, manganese ions are shown as purple spheres. (*B* and *D*) A 90° rotated “side” view of *A* and *C*.

To provide a detailed understanding of the changes in antibody binding modes enabled by the extensive affinity-maturation process, we set out to solve the crystal structure of the complex formed between the parent Fab C0020187 and ARG2, which would allow comparisons with the structures previously obtained for representative affinity-matured antibodies bound to ARG2 to determine their mechanism of inhibition (PDB ID codes 6SS2 and 6SS4). As discussed previously, the high-affinity complex of Fab C0020187 bound to trimeric ARG2 showed a surprising 1:3 stoichiometry (C0020187:ARG2) due to substantial negative cooperativity in antibody binding. A sample of this complex was purified by SEC prior to setting up crystallization trials; however, unexpectedly a 3:3 complex was found within the crystals obtained, perhaps reflecting the relatively high-protein concentrations required for crystallization. The crystal structure of the ARG2-Fab C0020187 complex was refined to 3.25 Å in space group *P* 6_5_ 2 2, with a final *R*/*R*_free_ of 0.30/0.36. For detailed data collection and refinement statistics, see *SI Appendix*, Table S2. The asymmetric unit contained a trefoil-shaped ARG2 trimer and three Fabs, which were bound to ARG2 on very similar binding sites (Fig. 7 *A* and *B*).

**Fig. 8.**
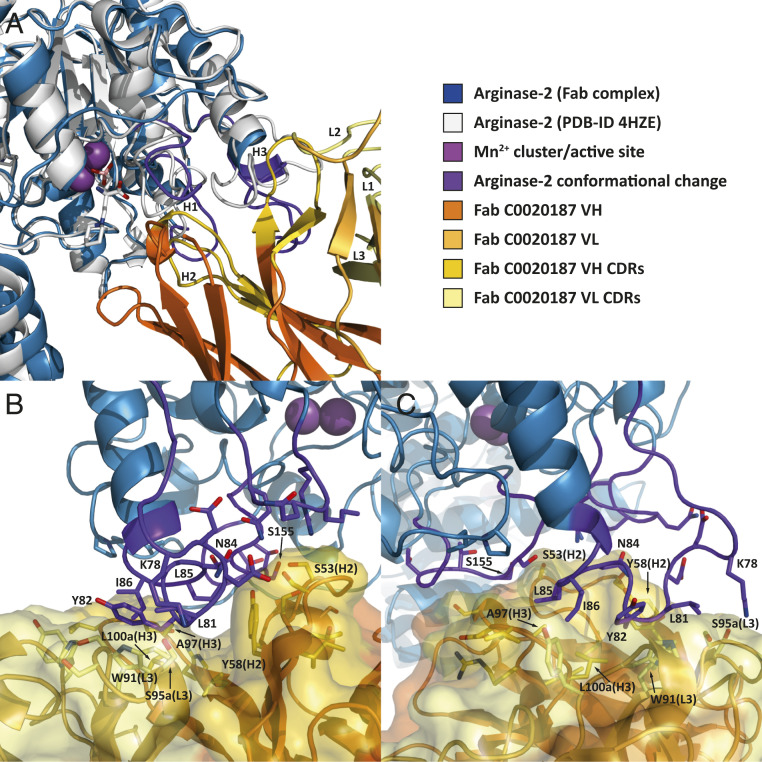
Close-up view of the binding interface between ARG2 and Fab C0020187. (*A*) Free ARG2 [PDB ID code 4HZE, light gray] was superimposed on Fab C0020187-bound ARG2 (excluding the regions of conformational change, shown in dark purple). The CDRs on the Fab are shown in yellow (VH) and light yellow (VL). (*B* and *C*) The 90° rotated side views of the binding interface between Fab C0020187 and ARG2, with interacting side chains shown as sticks. The Fab (VH/CH in orange and VL/CL in light orange, CDRs in yellow and light yellow for VH and VL, respectively) is shown as a cartoon inside a semiopaque surface representation. ARG2 is shown as a blue cartoon, with the region undergoing a major conformational change upon antibody binding highlighted in dark purple. Manganese ions are shown as purple spheres.

The parental Fab C0020187 binds to ARG2 close to the active site, but does not appear to sterically block access, which suggests an allosteric mechanism of action. Antibody binding induces a large conformational change in several neighboring regions of ARG2 (residues 34 to 40, 71 to 88, and 150 to 159) (Fig. 8*A*), with the first group of residues relatively close to the active site. The interface area between ARG2 and Fab C0020187 calculated by PISA (26) is about 654 Å^2^ when averaged over the three interfaces in the asymmetric unit (*SI Appendix*, Table S3). Most interactions are between the regions of conformational change in ARG2 (interface residues 35 to 39, 78 to 86, 152 to 157, and 178 to 179) and CDRH2/CDRH3 and CDRL1/CDRL3. Residues within CDRH3 and CDRL3 form a hydrophobic cleft on the antibody surface, but surprisingly residues forming the ARG2 epitope do not insert deeply (Fig. 8 *B* and *C*). Residues with the largest changes in accessible surface area on antibody-binding and located in the center of the interface include: ARG2 Y82 (van der Waals [vdW] interaction with VH L100a and π-stacking with VL W91), ARG2 L81 (vdW interaction with VL W91), ARG2 I86 (vdW interactions with VH A97 and VH L100a), and ARG2 L85 (vdW interaction with VH A97). There are also a number of strong polar interactions including H-bonds between ARG2 K78 and VL S95a, ARG2 N84 and VH Y58, and ARG2 S155 and VH S53 (Fig. 8).

Detailed comparisons of the crystal structures obtained for the parent Fab C0020187 and representative affinity-matured Fabs (C0021158 and C0021181) bound to ARG2 (PDB ID codes 6SS2 and 6SS4), reveal some striking and important differences in antibody binding, but also many similarities in the inhibitory conformational changes induced in ARG2 (see Movie S1 for a morphing movie illustrating the epitope shift between C0020187 and C0021158 and *SI Appendix*, Fig. S5 for a sequence-based comparison). While Fab C0020187 binds to the “sides” of the ARG2 trefoil (Fig. 7 *A* and *B*), the epitopes and orientation of Fabs C0021158 and C0021181 are dramatically shifted, with the Fabs being positioned “underneath” the ARG2 trefoil (Fig. 7 *C* and *D*), with substantially increased interface areas of 1014 Å^2^ and 910 Å^2^, respectively. The affinity-matured Fabs’ interfaces are thereby rotated by ∼120° compared to Fab C0020187. Interestingly, even though Fabs C0021158 and C0021181 do not contact the area around residues 152 to 157, they nevertheless cause conformational changes in the same regions as Fab C0020187, most notably the radical remodeling of the surface loop 71 to 88, but with an entirely different bound conformation. Fabs C0021158 and C0021181 additionally interact with residues 299 to 308 on ARG2, but without inducing a conformational change in that region. Even though the central hydrophobic cleft exhibits the same conformation in the parent Fab C0020187 and the affinity-matured Fabs (C0021158 and C0021181), the parent antibody does not induce the single-turn hydrophobic helix in ARG2, which is seen to fill the hydrophobic cleft between the VH and VL of the complexed affinity-matured Fabs. Most amino acid substitutions between the parent Fab C0020187 and affinity-matured Fabs (C0021158 and C0021181) are concentrated within CDR H1. This does not form part of the conserved central hydrophobic interface in the inhibitory ARG2-antibody complexes, but instead facilitates the interaction with residues 299 to 308 of ARG2 after affinity maturation. CDR H1 is therefore most likely responsible for the epitope shift, while the hydrophobic cleft interactions probably induce the major conformational changes in ARG2 driving the allosteric inhibition of activity.

## Discussion

Conventional affinity-maturation strategies are often restrictive, laborious, and slow. Iterative rounds of library building, selections, and screening are required, which are then followed by successive rounds of recombination and postrecombination screening. A large number of library builds are required to enable this process, and at each intermediate stage choices around which libraries are targeted for mutagenesis or used in subsequent recombinations are made based on the best available evidence at that time. In our affinity-maturation campaign, we have taken an unbiased approach to explore multiple avenues simultaneously, thus minimizing the chances of success being limited by conventional thinking and maximizing the experimental space explored during our optimization. Therefore, as well as delivering time savings throughout the process, we envisage that this approach could deliver antibodies that are more distantly removed from their parent in terms of biochemical properties. Indeed, through the optimization of all six CDRs and an unbiased recombination that connected the beneficial mutations, large sequence changes were translated into global structural changes, which offered new possibilities for finding the ultimate sequence combination to provide the optimal binding solution for a challenging and complex antigen.

In this study, all six CDRs of the parental antibody were affinity-matured and their sequences were incorporated into a single-recombination library impartially using the Shuffle and ShuffleStEP method. This method resulted in an unbiased library that included mutations represented from each CDR, randomly recombined with each other. Selections allowed for the most favorable combinations to emerge, without any dictation on our part with regards to specific pairings. This eliminated the need for repetitive screening rounds and the somewhat speculative predictions as to which combinations may produce the optimal synergy. The application of pool maturation to the top antibody variants provided an addition of further diversity to the intermediate panel. Rather than having to choose one lead, or build separate libraries for each lead, we were able to affinity-mature all seven leads in a single pool. The EP approach produced random mutations scattered across the length of the scFv constructs. Apart from introducing new mutations and diversity into the sequence pool, this process has unexpectedly shuffled the DNA of the top seven leads to create hybrids, providing an additional push in increasing our combinatorial diversity and resulted in significantly improved antibodies with affinities in the 100- to 300-pM range.

The optimized lead panel consisted of antibodies with a relatively large number of mutations across multiple CDRs, with 18 amino acid changes in antibodies C0021158 and C0021181. Considering the unprecedented nature and magnitude of epitope movement and reorientation achieved, as the cocrystal structures revealed, it is probable that this level of change was crucial in overcoming some key limitations of the parental clone, such as the negative cooperativity in binding of multiple antibodies to the ARG2 trimer. The use of Shuffle/ShuffleStEP and pool maturation allowed sufficient scope for evolution of the antibody sequence to occur to enable such changes, while preserving the original inhibitory and allosteric functions. Traditional recombination methods would likely struggle to produce the level of diversity required.

Despite the large epitope shift, during the evolution of the parent to the affinity-matured clones, the hydrophobic interactions mediated by CDRH3 and CDRL3 remained unchanged. The hydrophobic cleft is a key interaction and is likely to be important for the mechanism of action. We observed from our results that changes to CDRH3 were not tolerated in this antibody lineage and were almost completely out-selected from the Shuffle and ShuffleStEP libraries after two rounds of selections, illustrating the rapid elimination of unworkable mutations in this process. CDRH3 is the most surface-accessible hypervariable loop within the variable domains and tends to be the most involved in binding interactions with antigens (4, 27), which is why it is usually prioritized for optimization during affinity maturation. However, optimization of this region did not appear to benefit our parental antibody and so an optimization strategy that focused on CDRH3 would not have been successful. CDRL3 changes involved in hydrophobic interactions appeared to favor increased penetrance of the ARG2 loop into the cleft, while the main contact residues (VL W91, VL S95a) remained unchanged. Interestingly the delicate nature of these changes is emphasized by our observation that single-CDR optimizations targeting CDRL3 did not perform particularly well in the single-CDR screens and would not have been chosen for recombination under normal circumstances, but the inclusion of these clones within the Shuffle/ShuffleStEP recombination proved valuable because these were not only tolerated but preferable when combined with mutations in other CDRs.

This illustrates that it is important to consider mutations in the context of other mutations in the antibody, which highlights one main strength of our method, namely to select for synergy from the onset. Recombination libraries are normally built sequentially, starting with two CDRs and perhaps adding a third or a fourth only after several rounds of selections and screening. The decision on which CDRs to recombine is normally drawn from the performance of each library in the single-CDR screen, which may not necessarily be the best way to predict CDR changes that work well together and may exclude CDR changes that could give additive gains in affinity. By incorporating multiple CDR changes simultaneously in the starting library like in Shuffle and ShuffleStEP, this effectively explored opportunities for producing unpredictable synergy.

Based on the sequence and structural evidence, the clustering of affinity-selected mutations within CDRH1 appear to be largely responsible for mediating the reorientation of Fab relative to ARG2, which increases the interface area by about 1.5-fold. This highlights a merit of the unbiased approach for nontraditional choices of CDRs (such as CDRH1) to be mutated over more conventional choices, such as CDRH3. It is also intriguing that if our mutagenesis was based on and limited by inferences from the structural biology of Fab C0020187 binding to ARG2, we might have been tempted to focus entirely on optimizing the interactions at the hydrophobic cleft by heavily mutating CDRH3 and CDRL3, which is very unlikely to have achieved the same gains during optimization as a result of improved binding interactions.

To summarize, the unbiased and simultaneous targeting of multiple CDRs for mutagenesis in our global approach has allowed for significant gains in the functional characteristics of the optimized antibody. The unbiased and nonpresumptuous nature of the method has effectively overcome the limitations of the parental antibody through nonpredictable changes in antibody sequence. The in-solution stoichiometric observations from the SEC analysis and the biphasic binding curves from the Octet, which characterized the parent Fab C0020187:ARG2 interactions, indicate negative cooperativity, which is not ideal from a therapeutic standpoint. The affinity-matured leads do not have this issue, and they readily bound to ARG2 with the expected 3:3 stoichiometry and exhibited binding curves which are consistent with the classic 1:1 binding model (three equivalent sites per ARG2 trimer).

Considering the high level of sequence and combinatorial diversity that was required to move from our initial lead antibody to a highly optimized candidate therapeutic, the choice of ribosome display as our selection platform becomes all the more essential. Ribosome display is a cell-free system, which has a theoretical limit that is only constrained by how many ribosomes can fit into a selection, which in practical terms translates to about 10^12^ (9, 10). This provides a much larger sequence space, which can accommodate a much greater library size than most other display technologies. The Shuffle and ShuffleStEP libraries mostly consisted of clones that have mutations in two or three CDRs. A typical single-CDR selection output consists of about 10^3^ to 10^4^ variants, which would put it within the capacity of ribosome display after recombination. Moreover, the gradual and random mutational nature of ribosome display also adds to the diversity, by introducing additional mutations across the whole scFv construct spontaneously over rounds of amplification during selections and recovery.

Conceptually, the unbiased approach to affinity maturation is about maximizing the opportunities for antibody improvement by being inclusive of changes offered by each CDR, each combination, and each lead of interest. As we have seen, this may result in large sequence changes, which can be accompanied by large structural or functional changes. In recognition, we have endeavored to implement a functional screen at each stage of the affinity maturation process to ensure that the antibody’s inhibitory function is preserved. The result was a final lead antibody with enhanced inhibitory function via an improved mode of binding.

Our antibody optimization strategy shares some interesting parallels with other studies in the literature, which seek to explore and recombine mutations across an increased sequence space. In one study, DNA shuffling via the use of DNase I digestion followed by enzymatic ligation, was used to recombine mutations accumulated after rounds of EP selections (28). In another study, a method named “look-through mutagenesis” was used to increase the number of mutational residues in up to three CDRs, by restricting amino acid variants to nine representative members based on side-chain chemistry (29). Like these studies, our optimization strategy was devised out of a desire to maximize the level of diversity that can be interrogated, to find an optimal balance within the constraints of display capacity. In comparison, our method is less conservative and provided a larger scope in terms of level of diversity achieved.

The work reported here provides a striking exemplar of the substantial improvements in potential therapeutic antibody potency that can be achieved by using an unbiased optimization approach to explore the full potential of therapeutic leads. The sequential application of Shuffle or ShuffleStEP followed by pool maturation allows therapeutic antibody discovery and development to harness the vast sequence space made available by ribosome display, resulting in extensive and synergistic mutations across the antibody-binding domains. Uniquely, we have captured the dramatic effect of these sequence changes in the cocrystal structures with ARG2, which revealed a large paratope reorientation to enable improvements in binding and mechanistic function. The approach reported here promises a widely applicable step change in therapeutic antibody optimization.

## Materials and Methods

### Antibody Optimization through Shuffle and ShuffleStEP Recombination.

Sequences in the six CDR regions of C0020187 were randomized by Kunkel mutagenesis (30) and the resulting libraries were selected using phage display, essentially as previously described (7). Purified DNA from the selection outputs were used as template for Shuffle and ShuffleStEP recombination. The VH and VL regions were amplified, and the full-length scFv constructs were generated by recombinatorial PCR, resulting in a library of shuffled VH and VL sequences. The H2L2 library was generated similarly except that only the outputs of CDRH2 and CDRL2 were used. StEP recombination was carried out using modified conditions of methods described in Zhao and Zha (16). During this process, the DNA products of the VH/VL shuffle were used as templates in a reaction containing 2.5 U ThermoPrime DNA polymerase, 75 mM Tris⋅HCl pH 8.8, 10 mM (NH_4_)_2_SO_4_, 1.5 mM MgCl_2_, 0.01% Tween 20, and 0.2 mM dNTPs with 1 µM of forward and reverse primers. After initial denaturation, the reaction was taken through very short annealing/extension steps: 94 °C for 30 s/55 °C for 5 s for 80 cycles to promote cross-over events along the constructs. The resulting DNA was gel-purified and modified into ribosome display format using standard molecular biology methods.

### Ribosome Display.

Ribosome display selections were performed essentially as previously described (9). Stable ribosomal complexes with mRNA and translated scFv were incubated with biotinylated recombinant ARG2 (*SI Appendix*) overnight at 4 °C and binders were captured by streptavidin-coated paramagnetic beads (Dynabeads, Invitrogen). The mRNA was subsequently recovered, reverse-transcribed to cDNA, and then amplified by PCR. This DNA was used for the next round of selection and for subcloning into the pCANTAB6 phagemid vector for sequencing and bacterial expression as scFvs.

### Pool Maturation.

DNA constructs of the top seven leads identified in the screening cascade were purified and used as the starting template for pool maturation. EP mutagenesis was performed on the scFv template pool using the Diversify PCR random mutagenesis kit (Clontech) under conditions that result in 8.1 nucleotide changes per 1,000 bp according to the manufacturer’s instructions (9). The resulting DNA was gel-purified and converted into ribosome display format, then selected on biotinylated human ARG2 using ribosome display.

### EC Assay.

Assays were performed in buffer containing PBS, 0.1% (vol/vol) BSA and 0.4 M potassium fluoride. Test scFv were mixed with 3 nM biotinylated human ARG2, 1.67 nM streptavidin-cryptate, 10 nM anti-human Fc-XL665, and 4 nM C0020187 IgG in a total assay volume of 10 µL. After overnight incubation at 4 °C, fluorescence was measured at 665 nm and 620 nm, following excitation at 320 nm. Ratio values of (665/620 nm emission) × 10,000 were used to calculate Δ*F*% according to the following equation: Δ*F*% = [(sample ratio – negative control ratio)/negative control ratio] × 100. IC_50_s were calculated using GraphPad Prism software for titrated samples.

### EIA.

Test antibodies were preincubated for 2 h at room temperature with 0.4 or 1 µg/mL human ARG2, for unpurified or purified samples, respectively, prior to detection of activity via methodology described by Jung et al. (31).

### Affinity Determination.

Kinetic experiments were carried out at 25 °C with orbital shaking at 1,000 rpm on the OctetRED96 instrument with reagents diluted in kinetics buffer (PBS containing 0.01% BSA and 0.002% Tween 20) in black 96-well plates. Biotinylated human ARG2 was captured at 3 µg/mL for 3 min to ∼3 to 4 nm response on streptavidin sensors. Antibodies in Fab format were titrated as analyte, with an association and dissociation time of 300 s and 600 s, respectively. This was extended to 600 s and 2,400 s for high-affinity (*K*_D_ in subnanomolar range) interactions with slow off-rates. An empty reference sensor was used for background subtraction. Association and dissociation rate constants were calculated based on fitting to the 1:1 binding model (or the heterogeneous ligand model for C0020187) on the Octet Data Analysis software 10.0.

### Protein Structure Determination.

Data collection was performed at Diamond Light Source beamline i04-1, and data were processed and scaled with XDS (32) and merged using AIMLESS (33). The structure was then solved by molecular replacement in PHASER (34). For detailed methodology, see *SI Appendix*.

### Data Availability.

Atomic coordinates and structure factors of ARG2:Fab complexes have been deposited in the Protein Data Bank, www.rcsb.org, under the ID codes 6SS5 and 6SS6. All other data are included in the main text and *SI Appendix*.

## Supplementary Material

Supplementary File

Supplementary File
