# Supplementary material for: Extensive sequence and structural evolution of Arginase 2 inhibitory antibodies enabled by an unbiased approach to affinity maturation
Source: Proc Natl Acad Sci U S A. 2020 Jul 2;117(29):16949–60. doi: 10.1073/pnas.1919565117 (PMC7382286; doi:10.1073/pnas.1919565117)
Supplement: Supplementary File [file pnas.1919565117.sapp.pdf]

Supplementary data

Figure S1. ***Block mutagenesis library design.*** The amino acid residues in each CDR were mutated in 'blocks' of six residues each. Sequences are annotated according to Kabat (S1 ref. 1) numbering and the residues corresponding to the CDRs are highlighted in blue, with selected flanking Vernier residues in black. The residues randomised in each library are denoted with the symbol X.

CDRH1 (H1)

Position (Kabat)	27	28	29	30	31	32	33	34	35
	F	T	F	S	S	Y	A	M	S
**Lib H1B1**				X	X	X	X	X	X

CDRL1 (L1)

Position (Kabat)	24	25	26	27	27a	27b	28	29	30	31	32	33	34
	S	G	S	S	S	N	I	G	N	H	Y	V	S
**Lib L1B1**				X	X	X	X	X	X				
**Lib L1B2**						X	X	X	X	X	X		

CDRH2 (H2)

Position (Kabat)	50	51	52	52a	53	54	55	56	57	58	59	60	61	62	63	64	65
	A	I	S	G	S	G	G	S	T	Y	Y	A	D	S	V	K	G
**Lib H2B1**	X	X	X	X	X	X											
**Lib H2B2**					X	X	X	X	X	X							

CDRL2 (L2)

Position (Kabat)	50	51	52	53	54	55	56
	D	N	S	E	R	P	S
**Lib L2B1**	X	X	X	X	X	X	

CDRH3 (H3)

Position (Kabat)	93	94	95	96	97	98	99	100	100a	100b	100c	101	102	103
	A	R	L	R	A	D	L	G	L	Y	M	D	L	W
**Lib H3B1**		X	X	X	X	X	X							
**Lib H3B2**					X	X	X	X	X	X				
**Lib H3B3**								X	X	X	X	X	X	

CDRL3 (L3)

Position (Kabat)	89	90	91	92	93	94	95	95a	95b	96	97
	G	T	W	D	S	S	L	S	A	L	V
**Lib L3B1**	X	X	X	X	X	X					
**Lib L3B2**				X	X	X	X	X	X		
**Lib L3B3**						X	X	X	X	X	X

www.pnas.org/cgi/doi/10.1073/pnas.1919565117

Figure S2. ***Construction of the Shuffle and ShuffleStEP Libraries.*** The library building process is shown in this schematic. Populations of VH- and VL-optimised outputs were amplified and recombined to form the Shuffle library. StEP recombination was used to introduce additional recombination events and promote intra-chain recombination, forming the ShuffleStEP library. The mutations accumulated in the single-CDR selection outputs were hence recombined in the libraries of new constructs for potential synergistic effects.

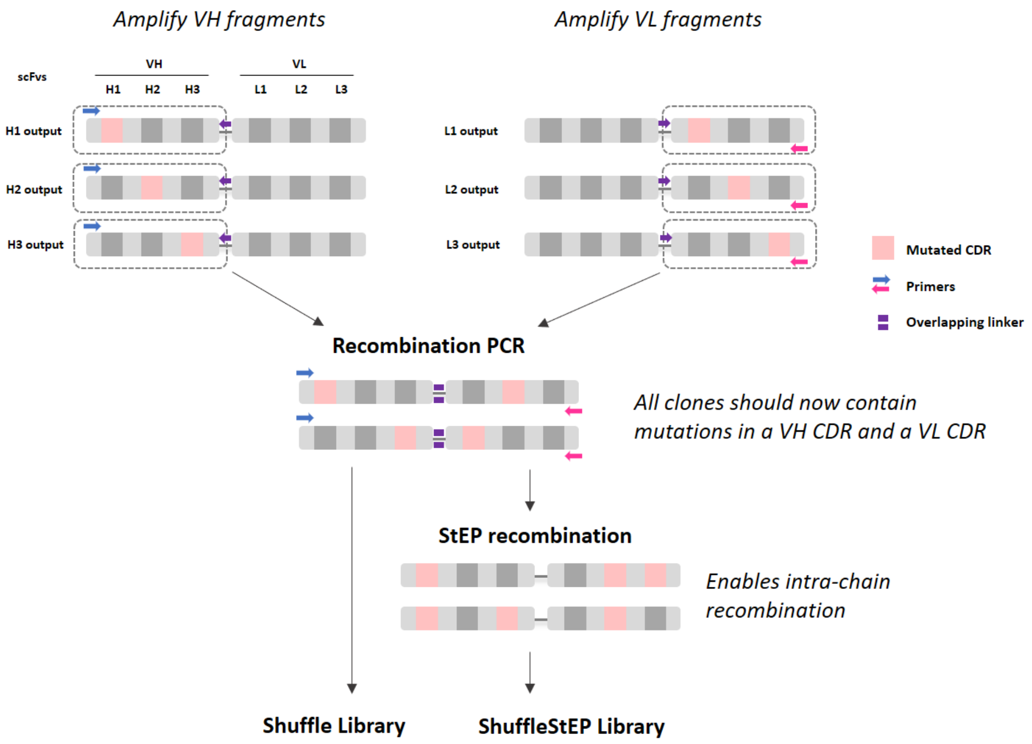

Figure S3: **Binding of the parent and affinity matured lead antibodies to human ARG2 (A)** Octet sensorgrams of the parent and lead antibodies in Fab format as analytes, with a 5-minute association and a 10-minute dissociation using a streptavidin capture assay. **(B)** Octet sensorgrams of lead antibodies C0021158, C0021177 and C0021181 in an extended Octet assay with 10-minute association and 40-minute dissociation. The concentrations (nM) of individual Fabs tested are shown below the relevant sensorgrams. Residuals from the fits are also shown under the sensorgrams. The sum of squared deviations (χ^2) is a measure of error between the experimental data and the fitted line.

(A)

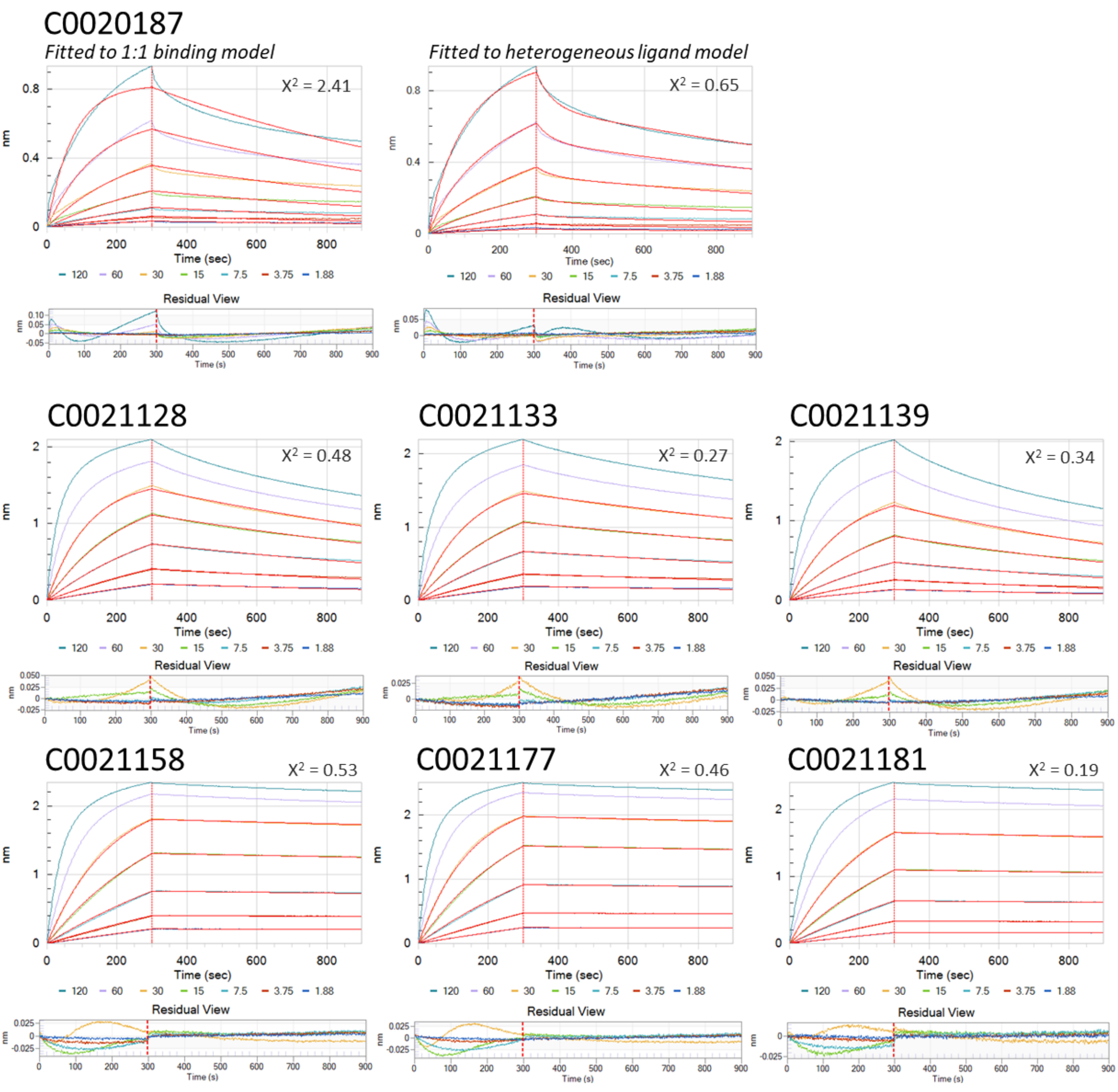

(B)

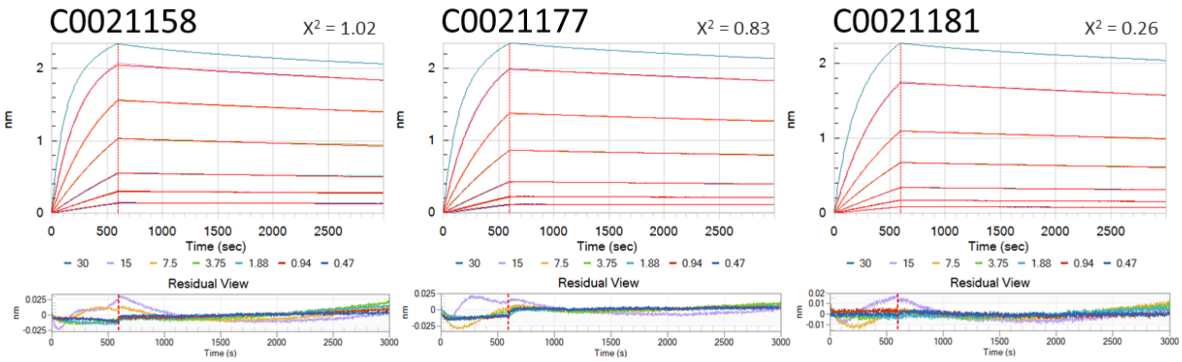
C0021158
X2 = 1.02
C0021177
X2 = 0.83
C0021181
X2 = 0.26
nm
Time (sec)
30
15
7.5
3.75
1.88
0.94
0.47
Residual View
Time (s)

Figure S4: **Size exclusion chromatography (SEC) analysis of complexes of human ARG2 with selected Fabs.** SEC traces of ARG2 alone, ARG2 complexed with Fab C0020187 (1:1.33 molar ratio) and ARG2 complexed with the affinity-matured Fabs C0021158, C0021177, C0021181 (1:1.15 molar ratio). The shift in retention volume compared to free ARG2 (13.0 ml) suggests that the parent Fab C0020187 shows high affinity binding to ARG2 in a 1:3 stoichiometry (11.8 ml), leaving a large excess of unbound Fab (16.5 ml), whereas the affinity-matured Fabs bind in a 3:3 stoichiometry (10.8 ml).

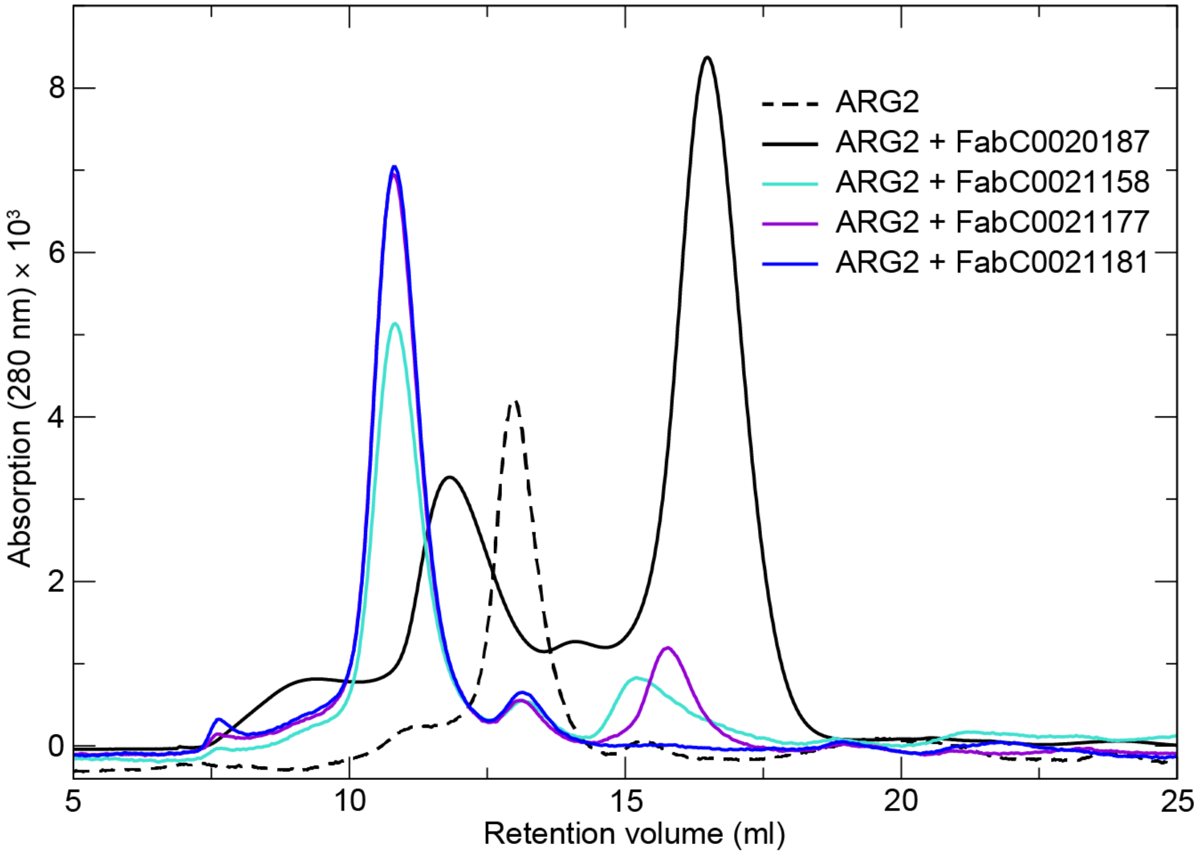

Figure S5. Summary of interactions between ARG2 and the Fabs C0020187 and C0021158. Compared to its parent (C0020187) the VH CDR1 residues of C0021158 form strong new interactions with different regions of ARG2 (Glu51, Pro299 and Gln300) and lose contacts with residues 152-157. Residue changes in CDR1 (and to a lesser extent CDR2) of the heavy chain seem to be the drivers that alter the binding mode towards ARG2. For C0020187, interactions are shown if they are present in at least one out of three ARG2-Fab interfaces and are consistent with at least one other. This variability arises as many side chains are not sufficiently well resolved at 3.25 Å.

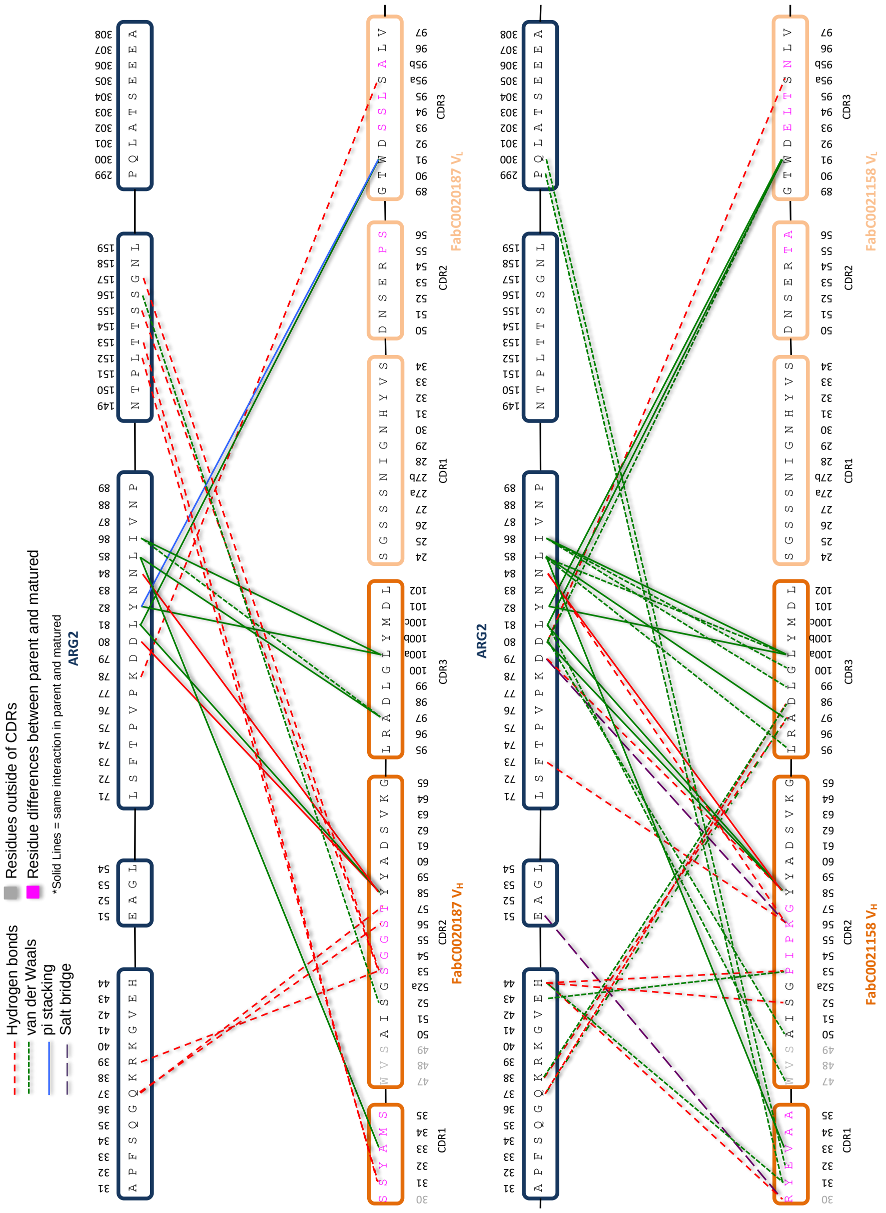

Table S1. ***Sequence analysis of the Shuffle and ShuffleStEP libraries.*** The amino acid sequences of clones sampled from the Shuffle and ShuffleStEP libraries were compared to the parental sequence to determine mutational and recombination frequencies. The percentage of clones in each mutational category was calculated by dividing the number of clones with mutations in x CDRs/number of clones sampled*100. Recombination frequency was defined as the percentage of clones in the population with recombined mutations in different CDRs (number of clones with aa mutations in more than one CDR / number of clones sampled*100). Percentage intra-chain recombination was calculated by dividing the number of clones with mutations in the same chain by the number of clones sampled*100.

	Shuffle Library	*ShuffleStEP Library*
**Percentage (%) of clones with mutations in:**		
0 CDR (parental sequence)	*0.0*	*2.5*
1 CDR	*9.9*	*16.3*
2 CDRs	*77.8*	*60.0*
3 CDRs	*7.4*	*18.8*
4 CDRs	*4.9*	*1.3*
5 CDRs	*0.0*	*0.0*
6 CDRs	*0.0*	*1.3*
**Recombination frequency (%)**	**90.1**	**81.3**
**Intra-chain recombination (%)**	**12.3**	**23.0**

Table S2: Data collection and refinement statistics. Values in brackets refer to the highest-resolution shell.

Structure	**Fab C0020187**	**ARG2 + Fab C0020187**
PDB ID	6SS5	6SS6
Space group	$C\ 2\ 2\ 2_1$	$P\ 6_5\ 2\ 2$
a (Å)	71.3	138.1
b (Å)	103.4	138.1
c (Å)	164.4	551.3
α (°)	90	90
β (°)	90	90
γ (°)	90	120
Resolution (Å)	49.31 – 1.78	48.78 – 3.25
Inner shell (Å)	(1.82 – 1.78)	(3.36 – 3.25)
R_{merge}	0.16 (>1)	0.78 (>1)
No. of observations	606016 (27795)	987566 (87473)
No. unique	58306 (3268)	50453 (4555)
Mean (I) / σ(I)	8.1 (0.5)	4.2 (0.4)
Half-set corr. CC(1/2)	1.00 (0.31)	0.99 (0.50)
Completeness (%)	99.7 (99.1)	100.0 (100.0)
Multiplicity	10.4 (8.5)	10.4 (9.9)
R / R_{free}	0.18 / 0.22	0.30 / 0.36
Bond RMSD (Å)	0.013	0.005
Angle RMSD (°)	1.78	1.46
Average B factors (Å^2)		

Main chain	32.6	107.9
Side chain	37.0	108.7
Ions and solutes	66.6	149.8
Waters	43.6	n.a.
Protein atoms[a]	3365	16814
Ion and solute atoms[a]	64	141
Water atoms[a]	318	n.a.
Ramachandran plot (%)		
Favored	96.2	93.1
Allowed	3.1	5.5
Outliers	0.7	1.4

[a] excluding hydrogen atoms

Table S3: Interface between ARG2 and Fab C0020187 as calculated using PISA. Values after the residues indicate in how many subunits the interaction was observed and over how many sites accessible surface area (ASA) and buried surface area (BSA) were averaged.

<table>
<tr><th colspan="2">ARG2 residue</th><th>H-bond/salt bridge</th><th>ASA (Å^2)</th><th>BSA (Å^2)</th><th>ΔG (kcal/mol)</th><th>% buried</th><th>Interface to Fab</th></tr>
<tr><td>GLN35</td><td>2/3</td><td>| |</td><td>32.3</td><td>13.7</td><td>-0.2</td><td>42%</td><td>V_H</td></tr>
<tr><td>GLY36</td><td>1/3</td><td>| |</td><td>23.8</td><td>0.3</td><td>0.0</td><td>1%</td><td>V_H</td></tr>
<tr><td>GLN37</td><td>3/3</td><td>|H|</td><td>118.7</td><td>24.4</td><td>-0.2</td><td>21%</td><td>V_H</td></tr>
<tr><td>LYS38</td><td>3/3</td><td>| |</td><td>170.6</td><td>36.0</td><td>0.3</td><td>21%</td><td>V_H</td></tr>
<tr><td>ARG39</td><td>2/3</td><td>|H|</td><td>65.8</td><td>21.4</td><td>-0.3</td><td>33%</td><td>V_H</td></tr>
<tr><td>LYS78</td><td>2/3</td><td>|H|</td><td>156.9</td><td>16.1</td><td>-0.1</td><td>10%</td><td>V_L</td></tr>
<tr><td>ASP79</td><td>1/3</td><td>| |</td><td>75.7</td><td>3.9</td><td>0.0</td><td>5%</td><td>V_H</td></tr>
<tr><td>ASP80</td><td>3/3</td><td>|H|</td><td>80.6</td><td>27.2</td><td>0.0</td><td>34%</td><td>V_H+V_L</td></tr>
<tr><td>LEU81</td><td>3/3</td><td>| |</td><td>109.1</td><td>85.8</td><td>1.0</td><td>79%</td><td>V_H+V_L</td></tr>
<tr><td>TYR82</td><td>3/3</td><td>| |</td><td>133.4</td><td>108.8</td><td>1.2</td><td>82%</td><td>V_H+V_L</td></tr>
<tr><td>ASN84</td><td>3/3</td><td>|H|</td><td>58.1</td><td>39.0</td><td>-0.3</td><td>67%</td><td>V_H</td></tr>
<tr><td>LEU85</td><td>3/3</td><td>| |</td><td>115.2</td><td>72.5</td><td>0.8</td><td>63%</td><td>V_H</td></tr>
<tr><td>ILE86</td><td>3/3</td><td>| |</td><td>74.8</td><td>47.9</td><td>0.8</td><td>64%</td><td>V_H+V_L</td></tr>
<tr><td>LEU152</td><td>3/3</td><td>|H|</td><td>123.8</td><td>23.7</td><td>-0.1</td><td>19%</td><td>V_H</td></tr>
<tr><td>THR153</td><td>3/3</td><td>| |</td><td>56.7</td><td>15.2</td><td>0.0</td><td>27%</td><td>V_H</td></tr>
<tr><td>THR154</td><td>3/3</td><td>|H|</td><td>45.0</td><td>14.7</td><td>0.1</td><td>33%</td><td>V_H</td></tr>
<tr><td>SER155</td><td>3/3</td><td>|H|</td><td>82.7</td><td>60.5</td><td>0.4</td><td>73%</td><td>V_H</td></tr>
<tr><td>SER156</td><td>3/3</td><td>|H|</td><td>51.9</td><td>21.8</td><td>0.1</td><td>42%</td><td>V_H</td></tr>
<tr><td>GLY157</td><td>3/3</td><td>|H|</td><td>35.6</td><td>22.2</td><td>0.2</td><td>62%</td><td>V_H</td></tr>
<tr><td>LEU178</td><td>1/3</td><td>| |</td><td>53.3</td><td>7.2</td><td>0.1</td><td>14%</td><td>V_H</td></tr>
<tr><td>PRO179</td><td>3/3</td><td>| |</td><td>72.3</td><td>16.7</td><td>0.3</td><td>23%</td><td>V_H</td></tr>
<tr><td>total:</td><td></td><td></td><td>1736.3</td><td>678.9</td><td>3.9</td><td>39%</td><td></td></tr>
</table>

<table>
<tr><th colspan="2">C0020187 VH residue</th><th>H-bond/salt bridge</th><th>ASA (Å^2)</th><th>BSA (Å^2)</th><th>ΔG (kcal/mol)</th><th>% buried</th></tr>
<tr><td>F27</td><td>1/3</td><td>| |</td><td>128.2</td><td>12.7</td><td>0.2</td><td>10%</td></tr>
<tr><td>T28</td><td>3/3</td><td>| |</td><td>83.7</td><td>4.6</td><td>0.1</td><td>5%</td></tr>
<tr><td>S30</td><td>3/3</td><td>|H|</td><td>48.8</td><td>13.3</td><td>-0.1</td><td>27%</td></tr>
<tr><td>S31</td><td>3/3</td><td>|H|</td><td>71.3</td><td>62.5</td><td>-0.2</td><td>88%</td></tr>
<tr><td>Y32</td><td>3/3</td><td>| |</td><td>55.8</td><td>8.6</td><td>0.0</td><td>15%</td></tr>
<tr><td>A33</td><td>3/3</td><td>|H|</td><td>29.5</td><td>20.4</td><td>0.3</td><td>69%</td></tr>
<tr><td>I51</td><td>1/3</td><td>| |</td><td>3.4</td><td>0.5</td><td>0.0</td><td>14%</td></tr>
<tr><td>S52</td><td>3/3</td><td>| |</td><td>25.2</td><td>18.9</td><td>0.3</td><td>75%</td></tr>
<tr><td>G52A</td><td>3/3</td><td>| |</td><td>13.0</td><td>9.0</td><td>0.1</td><td>70%</td></tr>
</table>

<table>
<tr><td>S53</td><td>3/3</td><td>|H|</td><td>102.9</td><td>53.1</td><td>0.2</td><td>52%</td></tr>
<tr><td>G54</td><td>2/3</td><td>|H|</td><td>23.9</td><td>6.4</td><td>0.0</td><td>27%</td></tr>
<tr><td>G55</td><td>3/3</td><td>| |</td><td>71.0</td><td>37.3</td><td>0.1</td><td>53%</td></tr>
<tr><td>S56</td><td>3/3</td><td>| |</td><td>40.7</td><td>22.5</td><td>0.0</td><td>55%</td></tr>
<tr><td>T57</td><td>2/3</td><td>|H|</td><td>60.7</td><td>7.9</td><td>-0.1</td><td>13%</td></tr>
<tr><td>Y58</td><td>3/3</td><td>|H|</td><td>128.2</td><td>97.1</td><td>0.8</td><td>76%</td></tr>
<tr><td>Y59</td><td>1/3</td><td>| |</td><td>41.3</td><td>0.2</td><td>0.0</td><td>0%</td></tr>
<tr><td>N73</td><td>1/3</td><td>| |</td><td>45.1</td><td>1.5</td><td>0.0</td><td>3%</td></tr>
<tr><td>L95</td><td>3/3</td><td>| |</td><td>28.0</td><td>5.4</td><td>0.1</td><td>19%</td></tr>
<tr><td>R96</td><td>2/3</td><td>| |</td><td>56.2</td><td>2.6</td><td>-0.1</td><td>5%</td></tr>
<tr><td>A97</td><td>3/3</td><td>| |</td><td>69.8</td><td>58.0</td><td>0.5</td><td>83%</td></tr>
<tr><td>D98</td><td>3/3</td><td>| |</td><td>84.5</td><td>8.4</td><td>0.0</td><td>10%</td></tr>
<tr><td>L99</td><td>3/3</td><td>| |</td><td>190.4</td><td>20.4</td><td>0.0</td><td>11%</td></tr>
<tr><td>G100</td><td>3/3</td><td>| |</td><td>40.1</td><td>10.4</td><td>0.1</td><td>26%</td></tr>
<tr><td>L100A</td><td>3/3</td><td>| |</td><td>139.1</td><td>47.5</td><td>0.7</td><td>34%</td></tr>
<tr><td>total:</td><td></td><td></td><td>1580.7</td><td>529.1</td><td>3.0</td><td>33%</td></tr>
</table>

<table>
<tr><th>C0020187 VL residue</th><th></th><th>H-bond/salt bridge</th><th>ASA (Å^2)</th><th>BSA (Å^2)</th><th>ΔG (kcal/mol)</th><th>% buried</th></tr>
<tr><td>H31</td><td>3/3</td><td>| |</td><td>40.9</td><td>19.2</td><td>0.2</td><td>47%</td></tr>
<tr><td>Y32</td><td>1/3</td><td>| |</td><td>141.7</td><td>1.4</td><td>0.0</td><td>1%</td></tr>
<tr><td>W91</td><td>3/3</td><td>| |</td><td>98.5</td><td>73.3</td><td>0.9</td><td>74%</td></tr>
<tr><td>S93</td><td>3/3</td><td>| |</td><td>53.7</td><td>9.8</td><td>0.0</td><td>18%</td></tr>
<tr><td>S94</td><td>1/3</td><td>| |</td><td>87.1</td><td>1.5</td><td>0.0</td><td>2%</td></tr>
<tr><td>S95A</td><td>3/3</td><td>|H|</td><td>81.1</td><td>35.8</td><td>0.0</td><td>44%</td></tr>
<tr><td>total:</td><td></td><td></td><td>502.9</td><td>141.0</td><td>1.1</td><td>28%</td></tr>
</table>

Movie S1. Morphing movie to illustrate the epitope shift between C0020187 and C0021158. A monomer of ARG2 (white) bound to Fab C0020187 (VH dark gray, VL light gray) is morphed into ARG2 (blue) bound to Fab C0021158 (VH orange, VL light orange, and CDRs yellow and light yellow, respectively). The morph was produced in PyMOL by coordinate interpolation and 3 subsequent rounds of geometry refinement. Fab C0020187 causes a slight extension of the N-terminal to ARG2 residue 88 and interacts with the residues 78-86 via its hydrophobic cleft between VH and VL. Fab C0021158 is rotated by approximately 120°, changing the epitope significantly, while still interacting with the same residues in its hydrophobic cleft between VH and VL. In this case, the Fab induces the formation of a short helix (ARG2 residues 81-85), which lies deep in the cleft.

A thumbnail of the movie is shown below.

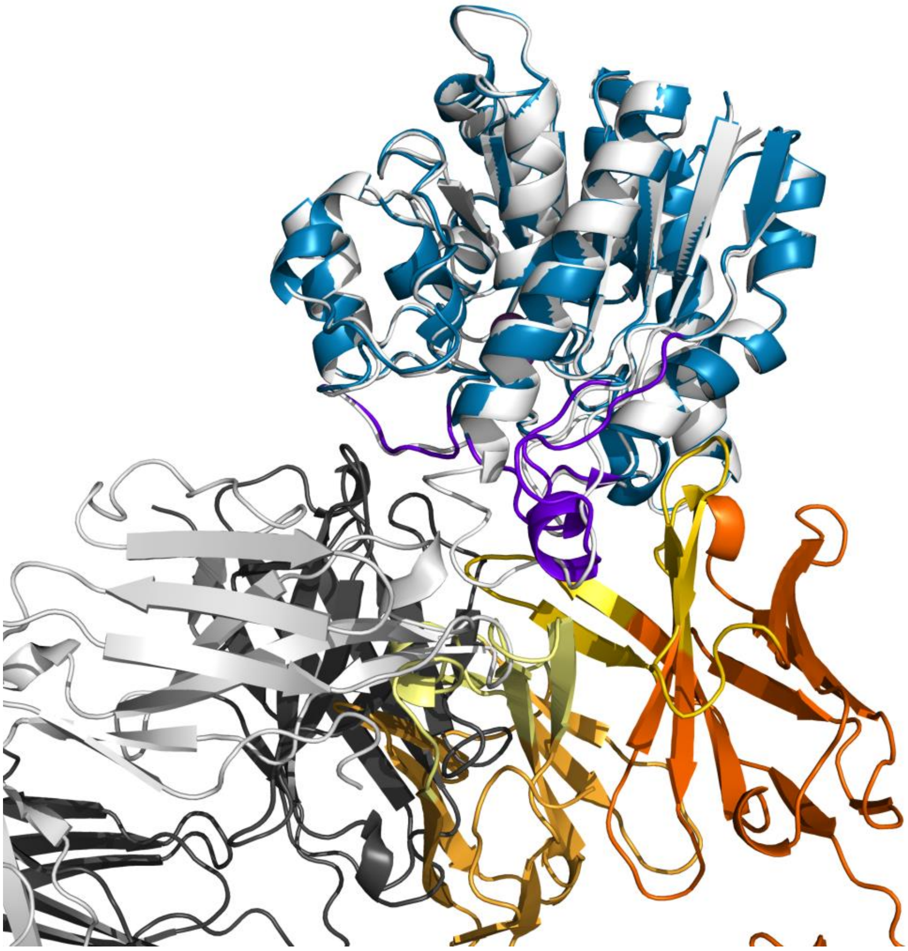

Supplementary Methods

Generation and biotinylation of trimeric recombinant ARG2

Expression vectors for ARG2 contained a synthetic DNA sequence encoding for human ARG2 (aa23-354) that had been codon optimised for expression in *E. coli*, plus flanking regions encoding for an N-terminal Avi tag and a C-terminal His10 tag. Amplified inserts containing the gene sequence of interest and tag(s) were cloned into pET16b vector (Novagen – EMD Millipore) using *Nco*I and *Xho*I restriction sites. The resulting vector was transformed into BL21 (DE3) *E. coli* and a single transformed colony was used to inoculate a culture of 2xTYA, which was grown overnight at 37 °C at 280 rpm.

The overnight culture was used to inoculate 2xTYA cultures in 2 L shake flasks. Each culture was incubated at 37 °C at 280 rpm until an OD600 of 0.6. The cultures were then moved to an incubator at 18 °C before the addition of IPTG (0.5 M), the resulting cultures were then incubated overnight at 18 °C at 280 rpm.

Cultures were pelleted by centrifugation at 12,000 rpm for 20 minutes at room temperature. The supernatant was decanted off and discarded before the pellet was frozen overnight at -80 °C. The resulting frozen pellet was thawed and processed for lysis using BugBuster™ Protein extraction reagent with the addition of lysonase bioprocessing reagent (Merck, 71370). Pellets were fully resuspended in BugBuster reagent at 4 °C before the addition of lysonase, after addition of lysonase the pellets were incubated at room temperature on a shaker platform at approximately 120 rpm. Lysis mixture was then pelleted by centrifugation at 20,000 rpm for 45 minutes at 4oC.

Soluble fractions were then decanted and combined into a clean bottle. Ni-NTA resin (pre-washed and equilibrated into Tris 50 mM pH 8, 300 mM NaCl) was then added to supernatant and left to bind at 4 °C for 1 hour with gentle mixing. After binding, the Ni resin was filtered from the supernatant by gravity filtration through a Pierce Centrifuge column (ThermoFisher, 89898). The captured resin was washed (Ni wash buffer, Tris 50 mM pH 8, 300 mM NaCl, 40 mM imidazole), and then eluted in Ni elution buffer (Tris 50 mM pH 8, 300 mM NaCl, 400 mM imidazole).

The elution fraction was further purified on a Hiload 16 60 superdex 200 prep grade SEC column on an AKTAxpress by manual injection into a 5 ml loop. The column was equilibrated in and elution was performed with Tris glycerol storage buffer (25 mM Tris pH8, 150 mM NaCl, 10% glycerol) and 1.5 ml fractions were collected across the entire volume of the elution. Fractions containing observable peaks were pooled and analysed by SDS-PAGE and peaks were compared to molecular weight markers to establish trimeric fraction(s).

Trimeric huARG2 protein was diafiltered into 50 mM Bicine pH 8.3 using a 10KDa MWCO Slide-a-lyser dialysis cassette. 2.5 μg of biotin ligase (BirA enzyme, Avidity LLC, stock concentration 3 mg/ml) was used per 10 nmol of protein substrate. The ARG2 protein was concentrated to a final protein concentration of 40 μM (calculation based on 39.8 kDa as each monomer contains an Avi-tag). BiomixB (Avidity LLC, 10x stock solution) was used at 1x concentration. 50 mM Bicine at pH 8.3 was used to adjust the reaction to the final volume.

The reaction was left to run at room temperature for 2 hours. The mixture was subsequently buffer exchanged into Tris glycerol storage buffer (25 mM Tris pH8, 150 mM NaCl, 10% glycerol) (pre-chilled to 4 °C) by overnight dialysis using a 10 KDa MWCO Slide-a-lyser dialysis cassette. Afterwards the biotinylated material was purified on a Hiload 16 60 Superdex 200 prep grade SEC column on an AKTA express by manual injection into a 5 mL loop. The column was equilibrated in and elution was performed with Tris glycerol storage buffer (25 mM Tris pH8, 150 mM NaCl, 10% glycerol) and 1.5 mL fractions were collected across the entire volume of the elution. Fractions containing the protein as compared to molecular weight markers (Alcohol dehydrogenase and Carbonic anhydrase – from Sigma gel filtration marker kit MWGF1000) were pooled.

Parent Fab C0020187 crystallisation, data collection and structure determination

The purified Fab was concentrated in TBS pH 7.4 to 16.5 mg/ml and pipetted as sitting drops (0.15 µl Fab + 0.14 µl reservoir solution using an Oryx8 robot) into JCSG+ and PACT screens. JCSG+ condition D5 (100 mM HEPES buffer pH 7.5, 70% MPD) yielded irregular, disc-shaped crystals of approx. 100 µm × 30 µm × 10 µm within less than a week at 18 °C. The crystals were cryo-cooled in liquid N_2 and data collection was performed at DLS beamline i04-1. One crystal diffracted to approximately 1.8 Å and indexed in space group $C\,2\,2\,2_1$. The data were processed and scaled with XDS (S1 ref. 2) and merged using AIMLESS (S1 ref. 3). The structure was then solved by molecular replacement in PHASER (S1 ref. 4) using a high-resolution structure of Fab C0021144 (PDB-ID: 6SRV, (Austin, M. & Burschowsky, D. et. al.; paper submitted to mAbs), and was subsequently refined using REFMAC5 (S1 ref. 5) and COOT (S1 ref. 6) (final R/R_{free} = 0.18/0.22).

ARG2/Fab C0020187 complex formation, crystallisation, data collection and structure determination

Human full-length ARG2 (residues 23-354, C-terminal Gly_3 linker and His_{10}-tag) and Fab C0020187 were mixed in a molar ratio 3:4 (12 µM:16 µM in TBS pH 7.4) and subjected to size exclusion chromatography on a Superdex 200 16/600 column into 20 mM Tris pH 8.0, 100 mM NaCl. Fractions with an apparent stoichiometry of ARG2:Fab C0020187 = 3:1 were pooled and concentrated to 2.0 mg/ml. The crystallisation was set up as sitting drops (0.15 µl complex + 0.14 µl reservoir solution using an Oryx8 robot) at 18 °C in Morpheus and SG-1 screens (Molecular Dimensions). The condition yielding the best-diffracting crystal contained 2 M $(NH_4)_2SO_4$. The crystals grew within two days to rounded, hexagonal disc shapes, with dimensions of approx. 60 µm × 60 µm × 20 µm.

After adding 3 M $(NH_4)_2SO_4$ and 5% glycerol for cryo-protection, the crystals were cryo-cooled in liquid N_2 and data collection was performed at the DLS macromolecular beamline i04-1, where a dataset diffracting to approx. 3.2 Å was obtained.

The data were processed and scaled with XDS (S1 ref. 2) and merged using AIMLESS (S1 ref. 3). The crystal indexed in space group $P\,6_5\,2\,2$. The structure was then solved by molecular replacement in PHASER (S1 ref. 4) using a high-resolution ARG2 structure (PDB-ID: 4HZE (S1 ref. 7)) and the high-resolution structure of the free Fab C0020187 (PDB-ID: 6SS5, this study) as search models. The asymmetric unit contained three copies of ARG2 and 3 copies of the Fab, with the CL and CH domains being present in different relative orientations. One single CH domain was poorly defined in the electron density, but was still found by PHASER with a high translation function Z-score (TFZ = 6.8) and log likelihood gain (LLG = 8737), indicating correct placement. Automated and manual refinement was then performed by alternatingly using REFMAC5 (S1 ref. 5) and COOT (S1.ref. 6), respectively. Refined R/R_{free} of the completed model were 0.30/0.36.

Acknowledgements:

The Cancer Research UK-AstraZeneca Antibody Alliance Laboratory is a long-term strategic alliance jointly supported by Cancer Research UK and AstraZeneca. The authors thank the Diamond Light Source for beam time under proposals mx14692 and mx19880, and especially thank the staff at beamline i04-1 for assistance with data collection. Parts of the study were funded by the Cancer Research UK Accelerator Award C1362/A20263.

Supplementary References:

1. G. Johnson, T. T. Wu, Kabat database and its applications: 30 years after the first variability plot. *Nucleic Acids Res* **28**, 214-218 (2000).
2. W. Kabsch, Xds. *Acta Crystallogr D Biol Crystallogr* **66**, 125-132 (2010).
3. P. R. Evans, G. N. Murshudov, How good are my data and what is the resolution? *Acta Crystallogr D Biol Crystallogr* **69**, 1204-1214 (2013).
4. A. J. McCoy *et al.*, Phaser crystallographic software. *J Appl Crystallogr* **40**, 658-674 (2007).
5. G. N. Murshudov *et al.*, REFMAC5 for the refinement of macromolecular crystal structures. *Acta Crystallogr D Biol Crystallogr* **67**, 355-367 (2011).
6. P. Emsley, B. Lohkamp, W. G. Scott, K. Cowtan, Features and development of Coot. *Acta Crystallogr D Biol Crystallogr* **66**, 486-501 (2010).
7. M. C. Van Zandt *et al.*, Discovery of (R)-2-amino-6-borono-2-(2-(piperidin-1-yl)ethyl)hexanoic acid and congeners as highly potent inhibitors of human arginases I and II for treatment of myocardial reperfusion injury. *J Med Chem* **56**, 2568-2580 (2013).
